# *Drosophila* hamlet mediates epithelial tissue assembly of the reproductive system

**DOI:** 10.7554/eLife.104164

**Published:** 2025-07-04

**Authors:** Huazhen Wang, Ludivine Bertonnier-Brouty, Isabella Artner, Jiayu Wen, Qi Dai

**Affiliations:** 1 Department of Molecular Bioscience, the Wenner-Gren Institute, Stockholm University Stockholm Sweden; 2 Lund University Lund Sweden; 3 Lund University Diabetes Center, Lund University Malmö Sweden; 4 Division of Genome Sciences and Cancer, The John Curtin School of Medical Research, The Australian National University, and Australian Research Council Centre of Excellence for the Mathematical Analysis of Cellular Systems Canberra Australia; Baylor College of Medicine United States; University of Barcelona Spain

**Keywords:** hamlet, PRDM factors, the reproductive system, epithelial tissue fusion, gene network, *D. melanogaster*

## Abstract

Epithelial tissue fusion requires coordinated molecular events at the ends of two epithelial structures. Regulatory mechanisms controlling these events remain largely elusive. In the *Drosophila* reproductive system (RS), this fusion unites the gonad and the genital disc-derived tissues, into a continuous tube. This study unveils the pivotal role of Hamlet (Ham), a *Drosophila* PR domain containing transcription factor, in orchestrating epithelial tissue fusion in the RS. Loss of *ham* leads to sterility and disconnection between the testes and seminal vesicles. Systematic analysis of Ham downstream genes reveals cytoskeletal, metabolic regulators and signaling pathway components. Ham activates genes for epithelial differentiation and remodeling, while repressing genes required for tissue growth and patterning. Using multiplexed in situ hybridization, we demonstrate spatial–temporal gene expression dynamics in contacting epithelia. Key Ham downstream effectors include E-Cadherin (E-Cad), Toll (Tl), and Wnt2 signaling pathways, regulating tissue interaction and fusion. Our findings present a comprehensive gene network crucial for heterotypic epithelial tissue fusion. Mammalian Ham orthologs PRDM3 and PRDM16 are highly expressed in epithelial tissues, suggesting a conserved role across species.

## Introduction

Epithelial tissue fusion is a crucial process for building epithelial tubular network and uniting separate ends (Bernascone et al., 2017; Chung and Andrew, 2008). Studies from different model systems, like neural tube fusion and *Drosophila* tracheal development, have provided several mechanistic insights into epithelial fusion process. For instance, specialized cells at the leading edge migrate and interact using cytoskeleton protrusions (Samakovlis et al., 1996a; Samakovlis et al., 1996b; Sutherland et al., 1996; Tanaka-Matakatsu et al., 1996; Gervais et al., 2012; Jacinto et al., 2001; Vasioukhin et al., 2000) and small GTPases like Rac and Cdc42 (Pai et al., 2012). Upon contact, the leading cells re-establish polarity and connections with the help of extracellular matrix molecules, apical proteins and E-Cad (Caviglia and Luschnig, 2014). However, the exact regulators and mechanisms controlling epithelial fusion remain to be determined, especially in a context-specific manner.

The reproductive system (RS) in animals comprises tissues from different origins, and must unite to form a connected system for producing and delivering gametes. For example, the human testis is directly linked to a complex tubular system consisting of the efferent and epididymal ducts (de Mello Santos and Hinton, 2019). Anomalies in the fusion of these ducts are a significant cause of cryptorchidism, affecting at least three percent of newborn boys. However, there is little understanding of the molecular mechanisms guiding the patterning and fusion of these ducts.

**Figure 1.**
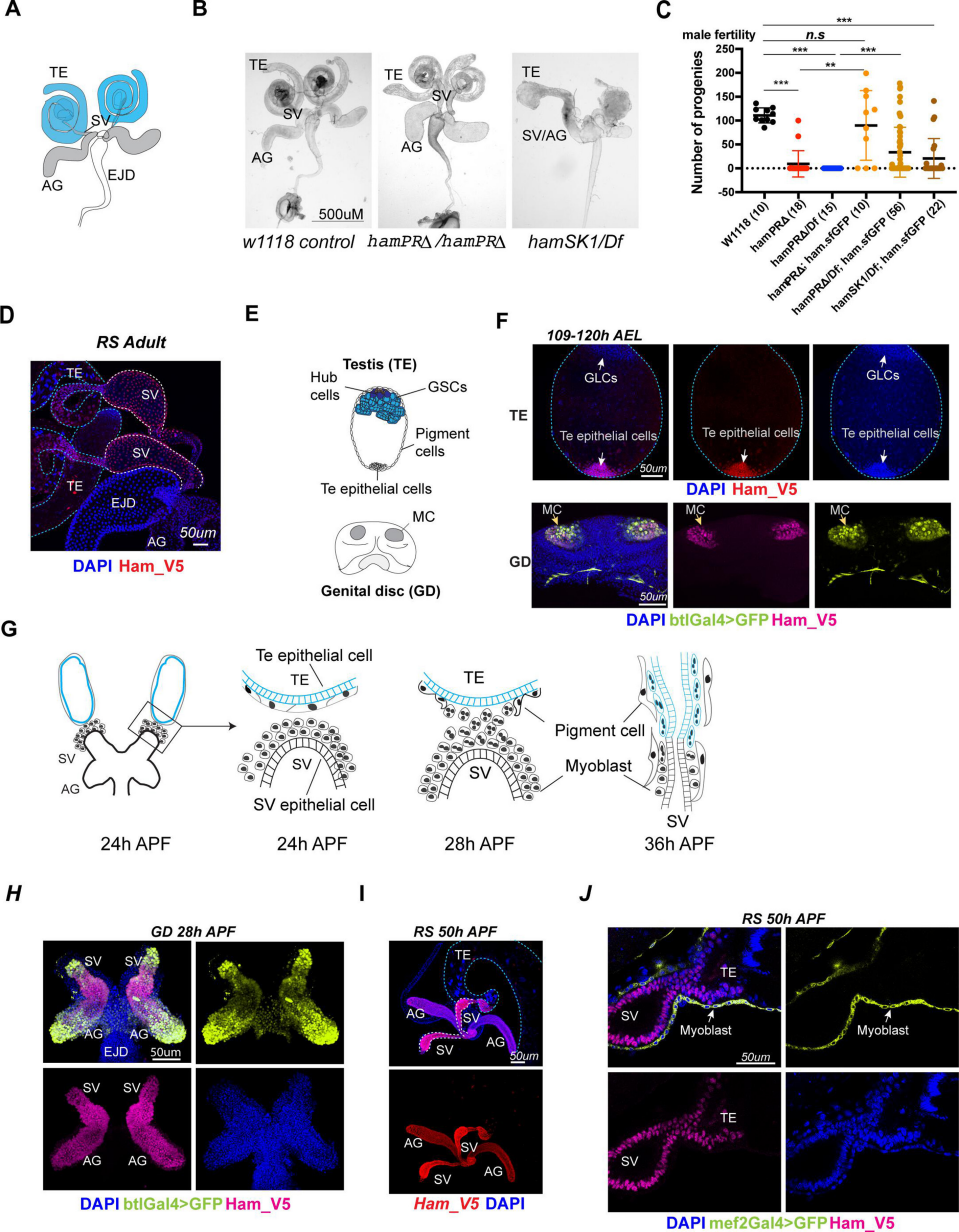
*ham* is expressed and required for *Drosophila* male reproductive system (RS) development. (**A**) The adult male RS. TE, testis; SV, seminal vesicle; AG, accessory gland; EJD, ejaculatory duct. (**B**) White-field images of adult male RS, showing defective morphology in the *ham* mutants. (**C**) Quantification of male fertility. Ham_sfGFP with the *ham* genomic region was used to rescue the sterility. Statistical significance was calculated using unpaired *t*-test (***p < 0.001; **p < 0.01; n.s., non-significant). (**D**) Images of the adult RS stained with Ham_V5 in red. DNA is stained with DAPI in blue. The blue and white dashed lines highlight the TE and SV, respectively. (**E**) Illustration of the developing male RS at the larval stage. The testis (TE) terminal epithelial cells localize at the opposite side of the germline stem cell (GSC) niche. The genital disc (GD) contains mesenchymal cells (MC) that will develop into the SV and accessory gland (ag). (**F**) Images of the larval TE (top) and GD (bottom) stained with Ham_V5 in red or magenta, the reporter in lime-green (btlGal4 driving UAS-GFP) and DAPI in blue. AEL, after egg laying. (**G**) Illustration of the TE and SV fusion process. APF, after pupal formation. (**H**) Images of the pupal GD stained with Ham_V5 in magenta, the reporter in lime-green (btlGal4 driving UAS-GFP) and DAPI in blue. (**I**) Images of the pupal RS with Ham_V5 in red and DAPI in blue. The blue and white dashed lines highlight the TE and SV, respectively. (**J**) Higher magnification image of the joint site between the SV and TE, with myoblasts and myotubes labeled by mef2Gal4>GFP in lime-green. Figure 1—source data 1.Raw counting data for the fertility test in Figure 1C.

Similarly, the *Drosophila* male RS (Figure 1A) includes the gonad-derived testis and the genital disc (GD)-formed seminal vesicle (SV), accessory gland (AG), ejaculatory duct (EJD), and additional supportive structures. During development, the TE and SV grow toward each other and merge into a continues tubular system (Jemc, 2011). This involves two epithelial ends, the terminal epithelial cells in the testis and the SV epithelium. Thus, the developing *Drosophila* RS provides a representative in vivo context to dissect mechanisms underlying epithelial tissue assembly.

**Figure 1—figure supplement 1.**
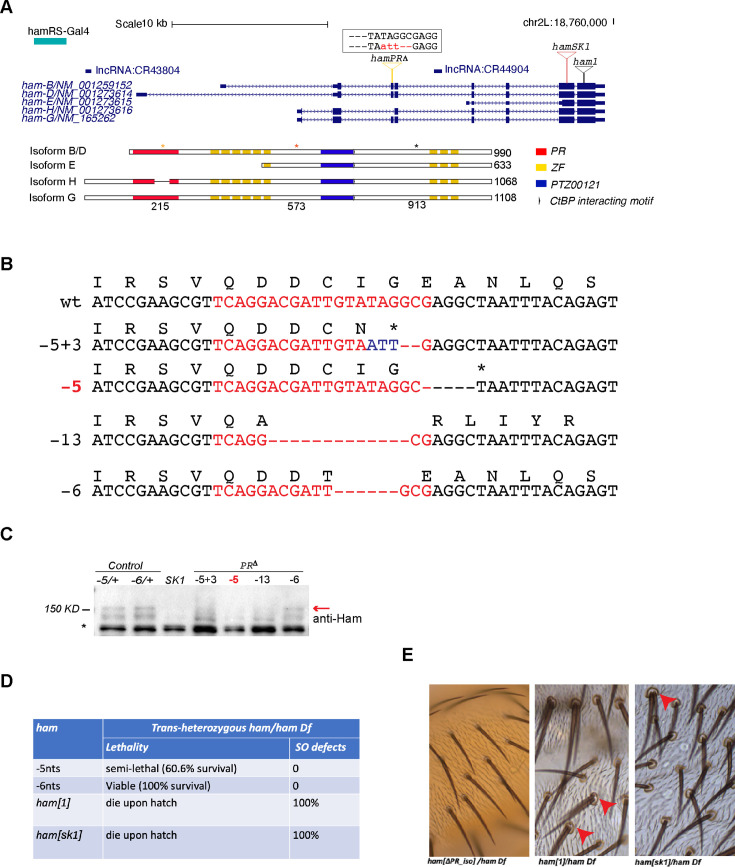
Ham gene locus and mutant alleles. (**A**) Illustration of the ham genomic locus and predicted protein isoforms, highlighting the mutation sites for the three mutant alleles and the protein domains of each isoform. All Ham protein isoforms maintain a functionally unknown motif PTZ00121 and the C-terminal zinc finger (ZF) clusters, while the long isoforms B/D and G also comprise the PR domain, the N-terminal ZF clusters. Isoform H has a truncated PR domain, and the shortest isoform E lacks the PR domain and the N-terminal ZF clusters. (**B**) Flanking sequences of the sgRNA target site from wt and four alleles with indels. Red letters indicate the sgRNA sequence. The corresponding amino acid sequence is shown above. ‘*’ indicates the stop codon. (**C**) An image from western-blot, showing the two Ham protein bands in the control samples and absence of the Ham band(s) in the indicated mutants. ‘*’ indicates an unspecific band, used as the loading control. (**D**) A table showing viability and sensory organ (SO) defects in four ham alleles. Note: the –6nt allele has a 6-nucleotide in-frame deletion. (**E**) Mechano-sensory organ phenotypes in the three indicated alleles. Red arrowheads indicate double sockets and double shafts phenotype. Figure 1—figure supplement 1—source data 1.This zip archive contains the raw unedited western blot shown in Figure 1—figure supplement 1C. Figure 1—figure supplement 1—source data 2.This zip archive contains the original western blot shown in Figure 1-figure supplement 1C with relevant band labeled.

**Figure 1—figure supplement 2.**
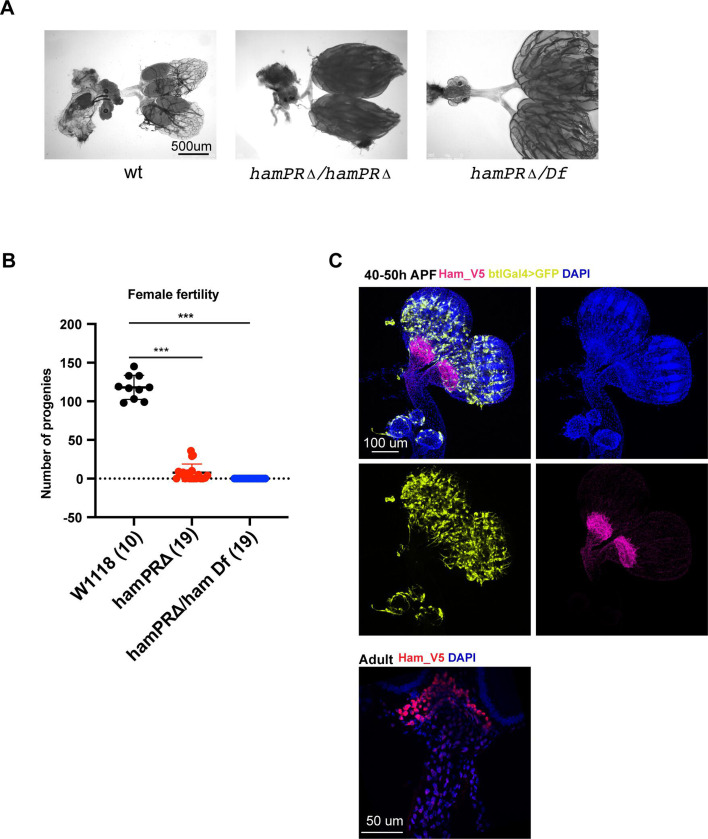
Ham expression and function in the female reproductive system (RS). (**A**) Bright-field images of the adult female RS from the indicated genotypes, showing accumulated eggs in the ham mutant ovaries. (**B**) Quantification of the number of progenies produced by wild-type and ham mutant females after crossed with wild-type males. Statistical significance was calculated using unpaired *t*-test (***p < 0.001). (**C**) Images of pupal and adult ovaries and oviducts, showing high expression of the Ham_V5 protein in the connecting sites of the ovaries and oviducts. Figure 1—figure supplement 2—source data 1.Quantification of female fertility shown in Figure 1—figure supplement 2B.

Ham belongs to the PR domain containing protein (PRDM) family whose members are evolutionarily conserved cell fate regulators (Fog et al., 2012; Chi and Cohen, 2016). The PRDM proteins are characterized by the presence of an N-terminal PR (PRDI-BF1 and RIZ1 homology) domain which is similar to the catalytic SET domain shared by histone methyltransferases. Dysfunction of the human orthologs of Ham, PRDM16 (Mel1) and PRDM3 (Evi1, MECOM), has been found in a variety of human abnormalities including congenital diseases, metabolic disorders and cancers (Bard-Chapeau et al., 2012; Zhou et al., 2016; Corrigan et al., 2018; Cibi et al., 2020; Kundu et al., 2020; Horvath and Scheele, 2022; Wang et al., 2022; Hurwitz et al., 2023). Moreover, in at least two types of epithelial cancers (kidney and pancreas cancers), PRDM16 was shown to play a tumor suppressive role (Kundu et al., 2020; Hurwitz et al., 2023), indicating its importance in maintaining epithelial integrity. However, it remains undermined how these PR factors regulate epithelial tissue development and homeostasis.

Ham was exclusively studied in the nervous system where it is required for neuronal dendrite morphogenesis (Moore et al., 2002) and cell fate maintenance (Moore et al., 2004; Rives-Quinto et al., 2020; Eroglu et al., 2014). Ham was shown to repress gene expression through modulating chromatin states (Endo et al., 2012) or interacting with the co-repressor CtBP (Moore et al., 2004; Endo et al., 2012). However, the molecular functions of PRDM16 and PRDM3 are versatile, with their PR domains possessing intrinsic histone methyltransferase activity (Zhou et al., 2016; Pinheiro et al., 2012) and the ability to interact with various co-activators and co-repressors (Fog et al., 2012; Hohenauer and Moore, 2012). It remains unknown whether Ham plays a role outside of the nervous system and whether Ham also has dual function in gene expression.

In this study, we discovered that Ham is a master regulator in guiding epithelial tissue patterning and fusion in the *Drosophila* RS. Even a slight reduction of Ham disrupts epithelial differentiation and tissue assembly in the RS, ultimately affecting sperm and egg delivery. To explore new mechanisms governing epithelial tissue fusion in the RS, we conducted systematic analyses using RNA-sequencing (RNA-seq), Cleavage Under Targets and Tagmentation (CUT&TAG), multiplexed in situ hybridization data and genetic screening. These analyses led to identification of gene regulatory networks required for epithelial tissue fusion. Ham activates a gene network to promote epithelial cell differentiation and remodeling while suppressing another gene network involved in cell specification and early patterning. These target genes exhibit spatial–temporal expression dynamics during the fusion of the TE and SV. As a key Ham downstream effector, Wnt2 signaling regulates epithelial cell communication and interaction. By elucidating the regulatory network controlled by Ham, our study provides novel insights into the molecular mechanisms underlying epithelial tissue fusion.

## Results

### *Drosophila* RS development is vulnerable to reduced Ham activity

While PRDM16 and MECOM are critical cell fate regulators in various developmental contexts in mammals, the genetic roles of Ham were mainly found in the central and periphery nervous systems (Moore et al., 2002; Moore et al., 2004; Rives-Quinto et al., 2020; Eroglu et al., 2014). To systematically study Ham function, we generated new frameshift alleles using CRISPR/Cas9. The sgRNA targets the third exon shared by the three long isoforms B, D and G (Figure 1—figure supplement 1A), producing new alleles specifically disrupting the three PR-domain containing isoforms. We therefore named them *ham^PRΔ^*. The original *ham^1^* and the null allele *ham^SK1^* shifted open reading frame (ORF) for all predicted isoforms (Moore et al., 2002; Rives-Quinto et al., 2020).

We validated nucleotide deletions in the new alleles by sanger sequencing (Figure 1—figure supplement 1B) and protein deletion by western blot using an antibody against Ham. The Ham antibody detected several bands in the blot (Figure 1—figure supplement 1C). The two bands at and above the 150 kD marker position in the two heterozygotic controls were not detectable in the *ham^SK1^* homozygous mutant, suggesting that these two bands correspond to the longest and the second longest isoforms, respectively. The *ham^PRΔ^* homozygous samples lack the largest band, verifying the depletion of the longest isoforms. We used one of them (–5nt) for all the later experiments.

We compared viability and sensory organ development of these alleles. In contrast to the original report showing that *ham* mutant animals died at the larval stage (Moore et al., 2002), we found that *ham^SK1^*, *ham^PRΔ^*, and *ham^1^* alleles all survived to hatch when combined in trans with the *ham* deficiency line (Df) which deletes the entire *ham* locus (Figure 1—figure supplement 1D). While *ham^1^*/Df and *ham^SK1^*/Df animals all died right after hatching, about two thirds of the *ham^PRΔ^*/Df mutants were viable without obvious gross defects. Furthermore, Ham is known to specify internal cell types (the neuron and sheath) in the mechano-sensory organ lineage, as *ham^1^* mutants exhibited ectopic external cell types (the socket and shaft) with the expense of internal cells (Moore et al., 2004). *ham^PRΔ^* mutants displayed normal sensory bristles, in contrast to defective sensory organs in the *ham^1^* and *ham^SK1^* alleles (Figure 1—figure supplement 1E). Thus, the PR-containing isoforms of Ham are dispensable for mechano-sensory organ development and viability.

However, *ham^PRΔ^* mutant animals showed abnormal RS morphology (Figure 1B) and impaired fertility in both male and female animals (Figure 1C, Figure 1—figure supplement 2A, B). The TE, AG, and SV structures were disrupted in males, while mature eggs were stuck in the mutant female ovaries, indicating failed egg delivery. To confirm that the defects are induced by the *ham* mutation, we performed a rescue experiment by utilizing a pBAC transgene that carries the genomic region of *ham* with the super-folding GFP (sfGFP) coding sequence in frame with the *ham* ORF. The pBAC transgene rescued the viability of all *ham* mutant conditions, and at least partly restored their male fertility (Figure 1C). Together, these results indicate that the *ham^PRΔ^* allele represents a hypomorphic condition which preserves Ham shorter isoforms and partial activity. Thus, although viability and sensory organ development do not require the PR-containing isoforms, formation of the RS depends on them.

### Ham-expressing epithelial cells connect the gonadal and supportive tissues

To assess how Ham controls RS development, we examined Ham expression in the developing RS using a V5 antibody which detects all Ham isoforms in the *ham_V5* allele (Gokcezade et al., 2014). Ham_V5 was detected in the connecting tube of the adult SV and TE (Figure 1D), suggesting a direct involvement of Ham in regulating RS formation.

During RS development, the TE terminal epithelial cells are specified in the embryo as male-specific somatic gonadal precursors (msSGPs) (DeFalco et al., 2004). The SV epithelium, together with AG, develops from a group of mesenchymal cells in the GD which undergo mesenchymal-to-epithelial transition (MET) during the larval stage (Figure 1E; Whitworth et al., 2012; Ahmad and Baker, 2002). Notably, in the L3 larval male RS, Ham_V5 was detected in the TE terminal epithelial cells as well as the btl-Gal4-expressing mesenchymal cells in the GD (Figure 1F).

In the pupal RS, multiple events, including epithelial tube formation and fusion as well as myoblast and pigment cell migration, are tightly coordinated (Figure 1G; Rothenbusch-Fender et al., 2017). At this stage, a high level of Ham persists in the TE terminal epithelial cells, SV and AG (Figure 1H–J). In contrast, Ham is barely detectable in the SV myoblasts/myotubes labeled by the reporter, mef2-Gal4-driving UAS-GFP (Figure 1J). Similarly, high levels of Ham_V5 were found at the joint sites of female oviducts and ovaries during both pupal and adult stages (Figure 1—figure supplement 2C). Thus, Ham is an intrinsic factor in epithelial cells at the connection site between the gonadal- and genital-disc-derived tissues in both males and females.

### Ham is required for epithelial tissue fusion in the developing male RS

The specific expression of Ham and mutant phenotypes in the RS prompted us to investigate its function in RS epithelial tissues. Here we focused on the male, as the assembly of the TE and SV represents a remarkable process involving three major cell types (myotubes, epithelial cells, and pigment cells) and a series of tissue reorganization events (Figure 1G). As the termini of the TE and SV approach each other, nascent myotubes—formed from myoblasts via cell–cell fusion at the SV surface—make contact with the TE terminus and begin to migrate onto the TE. Simultaneously, epithelial cells at the leading edges converge and fuse to form a continuous epithelial tube. Meanwhile, pigment cells from the TE migrate in the opposite direction. Ultimately, the myotubes organize into a thin muscular sheet, shaping the TE into a characteristic ‘spiral’ structure (Bischoff et al., 2021; Kuckwa et al., 2016).

**Figure 2.**
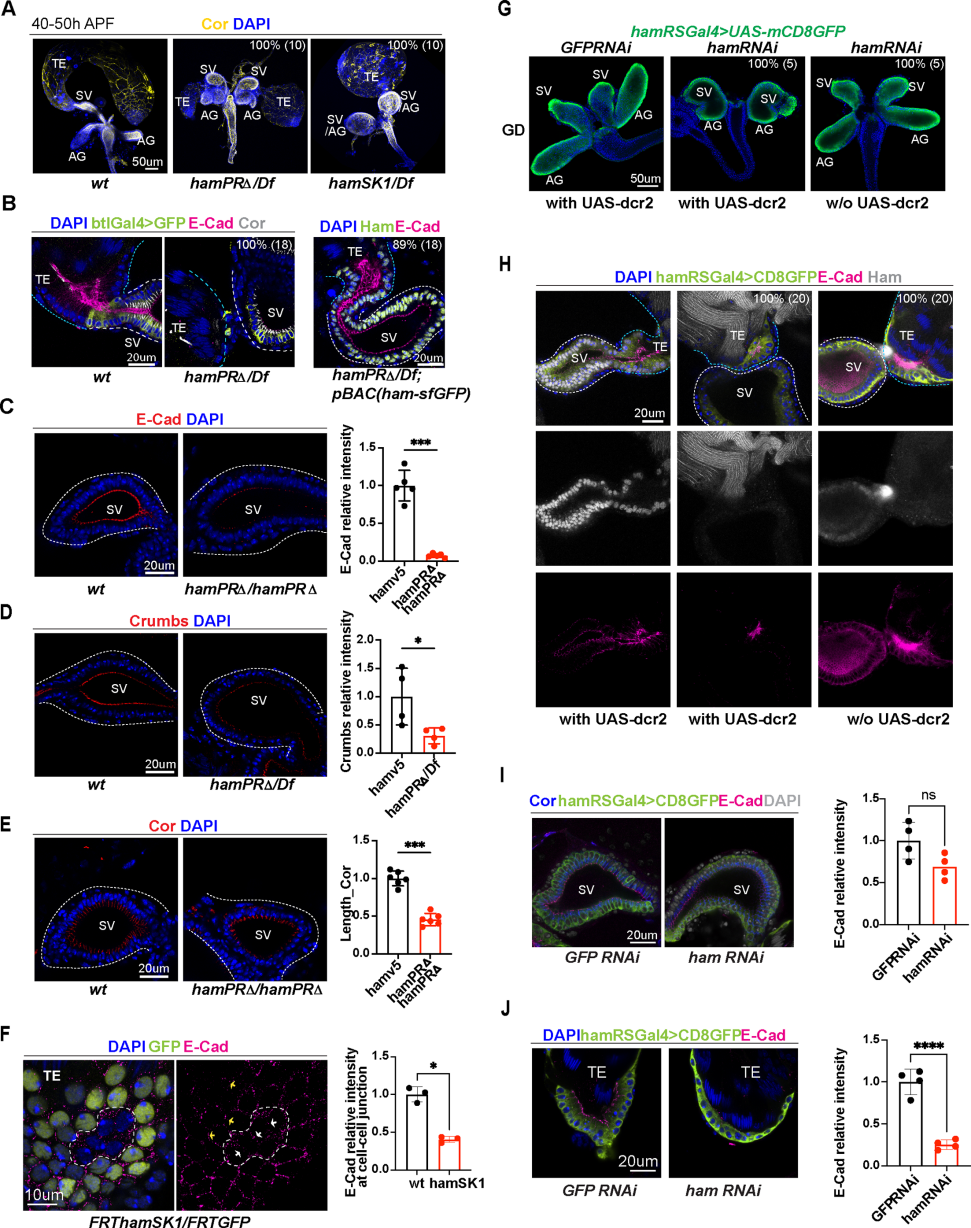
Ham promotes differentiation of the TE terminal and seminal vesicle (SV) epithelial cells. (**A**) Images of the male reproductive system (RS) at the pupal stage, showing morphological defects of the TE and SV in the ham mutants. Epithelial cells are labeled with Coracle (Cor). The phenotype penetrance (%) and number of animals (*n*) counted are indicated as % (*n*). (**B**) High magnification images of the connection site of the TE and SV, showing that the ham mutant RS failed to form a continuous tube. Ham-sfGFP restored the normal morphology. Epithelial cells are marked by E-Cad in red, Cor in yellow, and the epithelium on the SV side also marked by btlGal4 driving UAS-mCD8GFP. DNA is labeled by DAPI in blue. (**C**) Images and quantification of the E-Cad signal in the *wt* and *hamPRΔ* mutant SV. n = 5. (**D**) Images and quantification of the Crumbs signal in the *wt* and *hamPRΔ /Df* mutant SV. n = 4. (**E**) Images and quantification of the length of Cor signal in the wt and *hamPRΔ* mutant SV. n = 6. (**F**) Images of *ham* mutant mosaic clones in the TE terminal epithelial cells. Note, the mutant cells are GFP negative and within the area surrounded by the white dashed line. The white arrows indicate junctions between mutant cells, while the yellow arrows highlight the junctions between wild-type cells. n = 3. (**G**) Images of pupal male genital disc (GD), showing that *hamRNAi*-mediated knockdown reproduces *ham* mutant phenotypes. (**H**) Images of pupal male RS, stained with E-Cad in red, Ham in yellow, GFP in green and DAPI in blue. UAS-dcr2 enhanced knockdown efficiency and led to more severe morphological defects. (**I**) Images and quantification of E-Cad signal in red and Cor length in blue in control and ham RNAi SV. n = 4. (**J**) Images and quantification of E-Cad signal in control and ham RNAi TE terminal epithelium. n = 4. In the quantification graphs, statistical significance was calculated using unpaired *t*-test except for TE mosaic clones in F which used paired *t*-test (**** p < 0.0001, ***p < 0.001; **p < 0.01; *p < 0.05; ns, non-significant). Figure 2—source data 1.Quantification of the E-Cad signal in the *wt* and *hamPRΔ* mutant SV shown in Figure 2C. Figure 2—source data 2.Quantification of the Crumbs signal in the *wt* and *hamPRΔ /Df* mutant SV shown in Figure 2D. Figure 2—source data 3.Quantification of the length of Cor signal in the *wt* and *hamPRΔ* mutant SV shown in Figure 2E. Figure 2—source data 4.Quantification of the E-Cad signal at the border between two *wt* and between two *hamSK1* mutant cells in the TE terminal epithelium shown in Figure 2F. Figure 2—source data 5.Quantification of E-Cad signal in *GFP* and *ham*.*RNAi* SV shown in Figure 2I. Figure 2—source data 6.Quantification of E-Cad signal in *GFP* and *ham*.*RNAi* TE terminal epithelium shown in Figure 2J.

We found that at 40–50 hr APF the TE and SV tubes in *ham^PRΔ^/Df* and *hamSK1/Df* mutants did not properly elongate and appeared shorter and wider compared to that in the wild-type (Figure 2A). The mutant TE appeared as a sphere shape, indicating failed myotube migration. The SV and AG were only partly elongated in the *ham^PRΔ^* mutant, and even unseparated in the *ham* null allele *hamSK1/Df*. This suggests that the severity of RS abnormality correlates with the levels of reduction in *ham* gene products.

**Figure 2—figure supplement 1.**
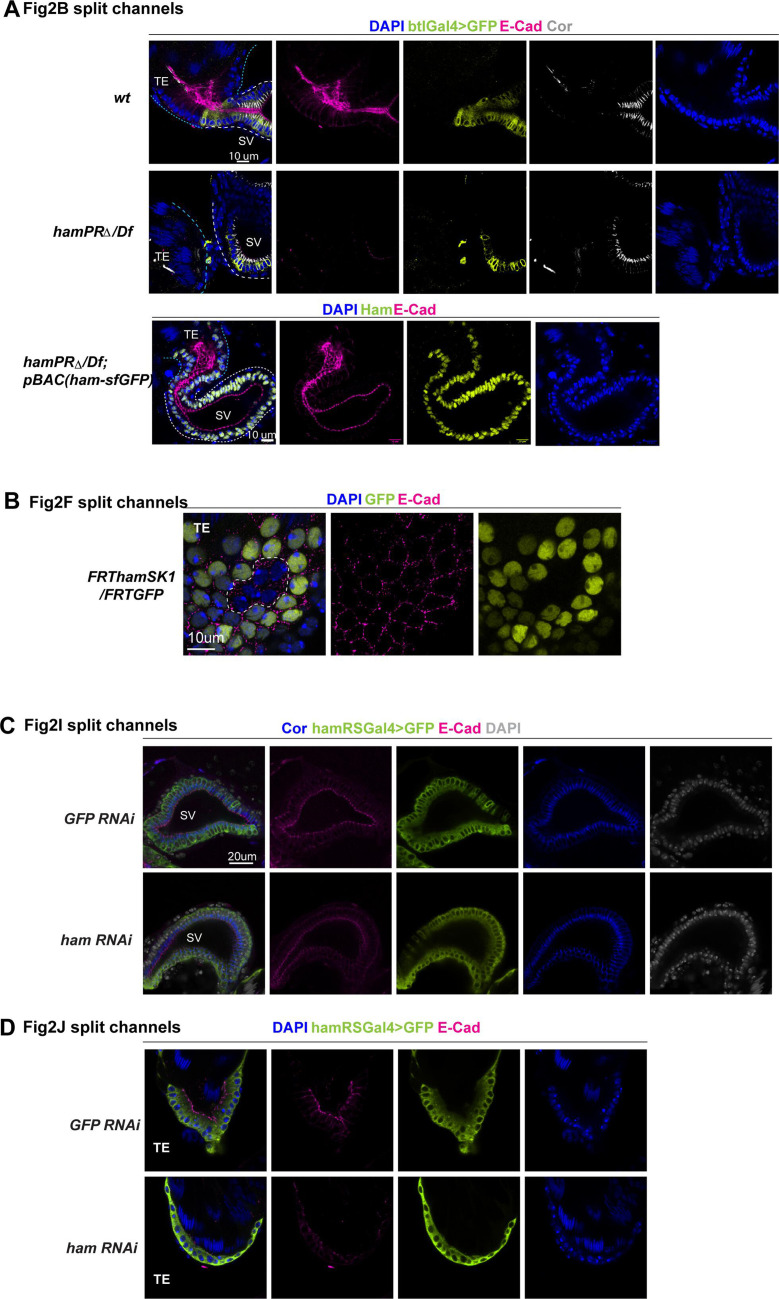
Split channel images for images in Figure 2.

**Figure 2—figure supplement 2.**
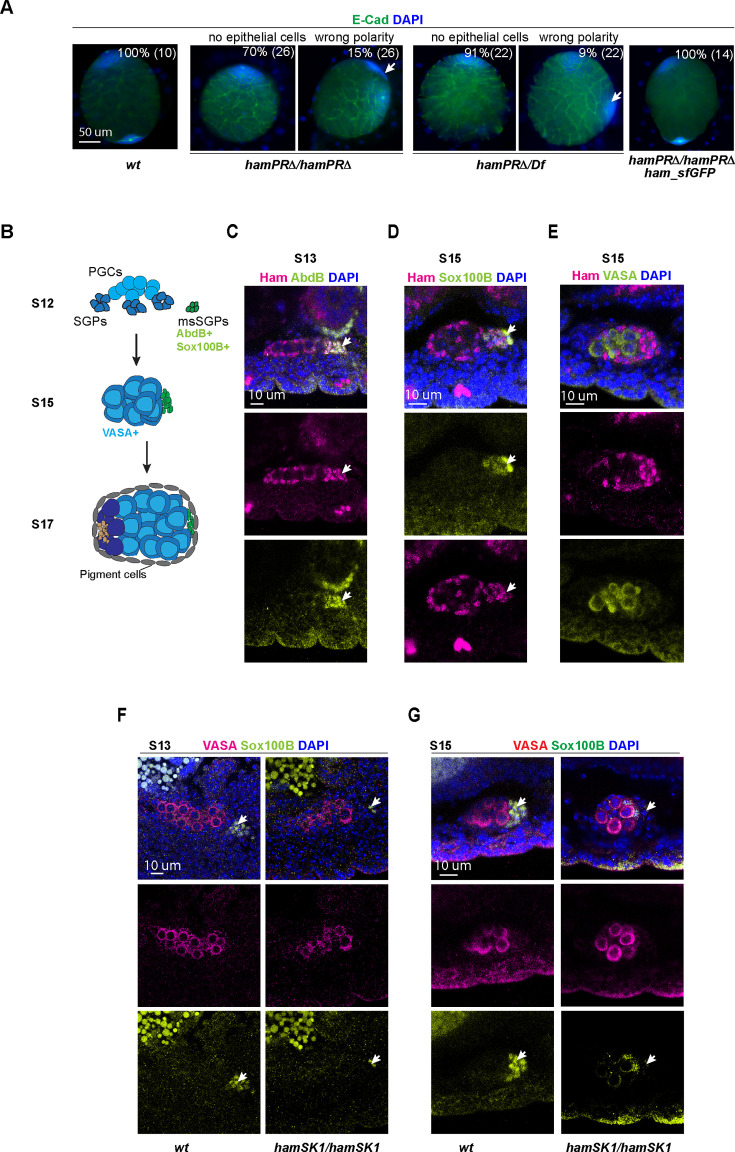
Ham controls the formation of TE epithelial cells. (**A**) Images of the testes from the early L3 larval stage, showing the absence of or mis-polarized TE terminal epithelial cell cluster in the *ham* mutant. E-Cad marks epithelial cells in green and DAPI marks the nuclei. The white arrows indicate mis-polarized epithelial cells. (**B**) Illustration of the precursor cell clusters of the male reproductive system (RS) in the embryonic stages. PGC, primordium germ cells; SGPs, somatic gonadal precursors; msSGP, male-specific SGPs. (**C–E**) Images of stage S13 and S15 *wt* embryos, showing Ham staining in all SGPs. Abd-B and Sox100B label msSGPs, VASA labels PGCs. Images of the *wt* and *ham* mutant embryos at S13 (**F**) and S15 (**G**), showing the reduction or absence of msSGPs in the *hamSK1* mutant. Vasa in magenta is a germ cell marker, and Sox100B labels msSGPs in lime-green.

**Figure 2—figure supplement 3.**
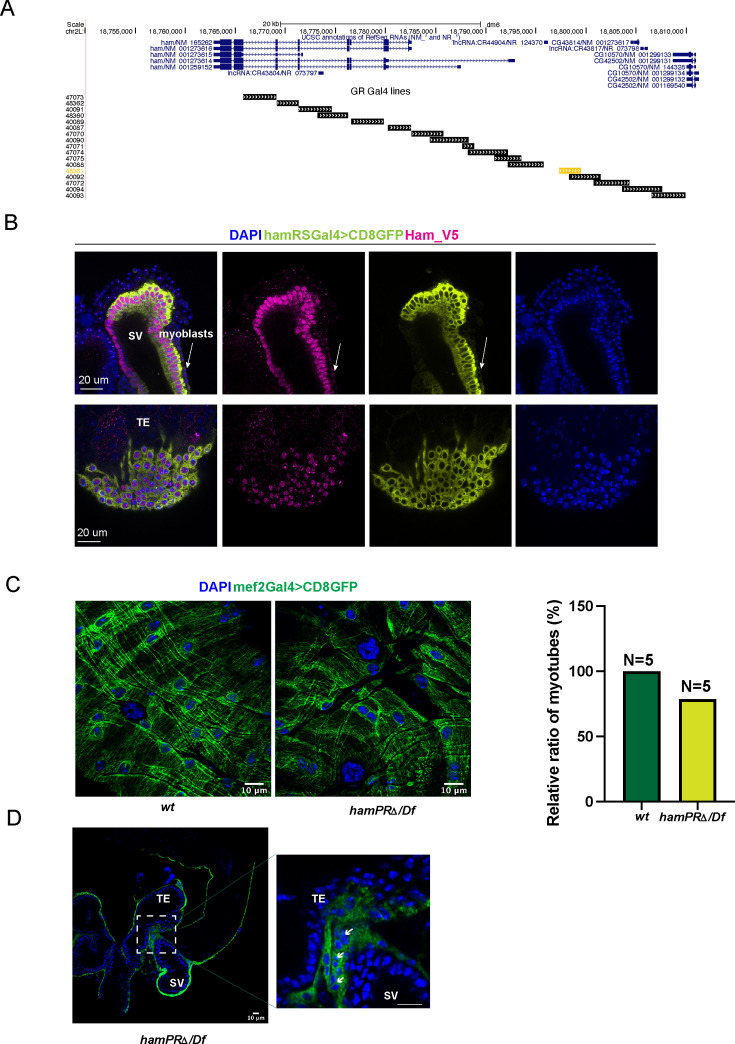
An epithelial-specific Gal4 line in the ham locus. (**A**) The *ham* gene locus, indicating the fragments in the Gal4 transgenes. The one (hamRSGal4) that drives reporter gene expression in the reproductive system (RS) epithelial cells is highlighted in orange. (**B**) Images of pupal genital disc and TE in animals with hamRSGal4 and UAS-mCD8GFP. Ham_V5 is stained in magenta, and DAPI in blue. (**C**) Images showing the presence of myotubes on the surface of the testis. The bar graph on the right shows the relative abundance of myotubes on the testis in wt (100%) and ham mutant condition quantified from five animals each. (**D**) Images showing the junction between the TE and seminal vesicle (SV), and myotubes are present even in the *ham* mutant. The white arrows indicate myotubes with two or three nuclei at the junction. Figure 2—figure supplement 3—source data 1.Quantification of myotube number on the testis in *wt* and *hamPRΔ/Df* shown in Figure 2—figure supplement 3C.

To examine epithelial integrity, we performed immunofluorescent staining using the antibodies against the epithelial cell markers, E-Cad (adherent junctions), and Coracle (Cor, septate junctions equivalent to mammalian tight junction) protein. We also incorporated an SV-specific transgenic reporter, btlGal4-driving UAS-EGFP or UAS-CD8GFP, to the control and *ham* mutant genotypes. E-Cad is enriched at the apical side (the lumen side, Figure 2B) of the TE and SV epithelial tubes. Notably, the TE terminal epithelium accumulates a higher level of E-Cad compared to the SV epithelium, while Cor is mainly expressed at the SV side but not much in the TE, suggesting a heterotypic nature of the TE terminal and SV epithelia.

Control animals always showed connected TE and SV, whereas ham mutant TE and SV tissues were either separated from each other, or appeared contacted but only with disconnected epithelial tubes (Figure 2B, Figure 2—figure supplement 1A). Importantly, this phenotype can be fully rescued by the *pBAC_ham_sfGFP* transgene, confirming that it is caused by the *ham* mutation. These observations suggest that the epithelial ends of the TE and SV possess distinct properties despite both being epithelial cell types and that Ham is required for TE and SV fusion.

### Depletion of Ham abolishes TE terminal epithelial cell formation

A close inspection on the pupal TE revealed that most of TE epithelial cells were missing in *ham* mutant animals (Figure 2B, Figure 2—figure supplement 1A). *ham* mutant lava also either lacked TE epithelial cells or showed abnormal location of these cells, which can be rescued by the *pBAC_ham_sfGFP* transgene (Figure 2—figure supplement 2A). TE terminal epithelial cells derive from male specific gonadal precursors (msSGPs) specified in the early embryo (Figure 2—figure supplement 2B; Whitworth et al., 2012) and expand during larval and early pupal stages. This result indicates that Ham regulates specification or survival of these cells. Indeed, we detected Ham expression in msSGPs and other SGPs in the embryonic gonad. The number of msSGPs labeled by Abd-B or Sox100B-GFP was dramatically reduced in S13 and S15 *ham* mutant embryo (Figure 2—figure supplement 2C–G). Our data suggest Ham to be a master regulator for the specification and/or survival of msSGPs, and the absence of msSGPs accounts for loss of TE terminal epithelial cells in larval and pupal stages.

### Ham regulates patterning and cell differentiation of the RS epithelial tubes

The epithelial cells in the SV and AG originate from a group of mesenchymal cells recruited by the FGF pathway involving Btl and Branchless (Bnl) (Whitworth et al., 2012; Ahmad and Baker, 2002). These cells undergo MET to form an epithelial mass which then cavitates to generate inner hollow space and eventually elongates into an epithelial tube. In comparison with the control, the signal intensity of both E-Cad and Crumbs (Crb) were significantly reduced in *ham* mutant SV epithelial cells, which coincides with the shortening of septate junctions labeled by Cor (Figure 2C–E). This result suggests that Ham regulates epithelial cell differentiation in the SV. To check whether Ham regulates epithelial cell differentiation in the TE, we used the FRT/FLP system (see Methods) to generate mosaic somatic mutant clones. In these animals, *ham* mutant cells were GFP negative, while wild-type and *ham* heterozygous cells expressed GFP (Figure 2F, Figure 2—figure supplement 1B). The E-Cad levels at the border between two mutant cells were significantly lower than those between wild-type cells, indicating that *ham* depletion impaired differentiation of TE terminal epithelial cells.

In summary, these phenotypic analyses suggest that Ham controls formation of TE terminal epithelial cells in the early embryo and regulates epithelial tube elongation and cell differentiation at the pupal stage. Thus, failed epithelium fusion in *ham* mutant RS could result from: (1) absence of TE terminal epithelial cells, (2) defects in epithelial tube elongation and cell differentiation of the SV, and (3) a combination of both.

### Ham directly controls epithelial fusion between the TE and SV

Next, we sought to assess whether and how Ham directly regulates the fusion process. We first attempted to generate a mild mutant condition which can bypass defects of TE epithelial cell loss and SV–AG tube elongation. We chose RNAi knockdown because knockdown efficiency can be enhanced by overexpressing Dicer2 (Dcr2) and modulated by temperature when RNAi is controlled by the GAL4/UAS system.

We first identified a Gal4 driver expressed in the SV and AG epithelial cells, after screening the available Gal4 transgenes driven by a series of genomic fragments spanning the *ham* locus (Jenett et al., 2012; Figure 2—figure supplement 3A). This transgene (thereafter named as *hamRSGAL4*) induces high expression of *UAS-GFP* in the TE and SV epithelial cells and a weak expression in the myoblasts (Figure 2—figure supplement 3B). Myotubes appeared at the junction site of the TE and SV and could migrate to the surface of the testis in the *ham* mutant (Figure 2—figure supplement 3C, D), confirming that loss of *ham* does not abolishes myoblast fusion or myotube migration. *hamRSGAL4*, when driving a *ham* RNAi line, nearly abolished Ham expression in animals carrying the *UAS-dcr2* transgene (Figure 2G, H, the middle panels). These animals also had a reduced number of TE epithelial cells and displayed a ball-like structure of the GD, similar to the *ham* null allele. Without *UAS-dcr2*, *ham* RNAi knockdown reduced Ham expression but preserved TE epithelial cells and normal-appearing SV. Remarkably, the TE and SV epithelia still failed to fuse (Figure 2H, the right panel), suggesting that Ham also controls the late step of fusion. Furthermore, E-Cad staining intensity was significantly reduced in *ham* RNAi TE but not in *ham* RNAi SV (Figure 2I, J, Figure 2—figure supplement 1C, D), suggesting that expression of E-Cad in the TE is sensitive to even mild reduction of Ham protein levels.

### Ham controls distinct molecular pathways to promote tissue assembly

**Figure 3.**
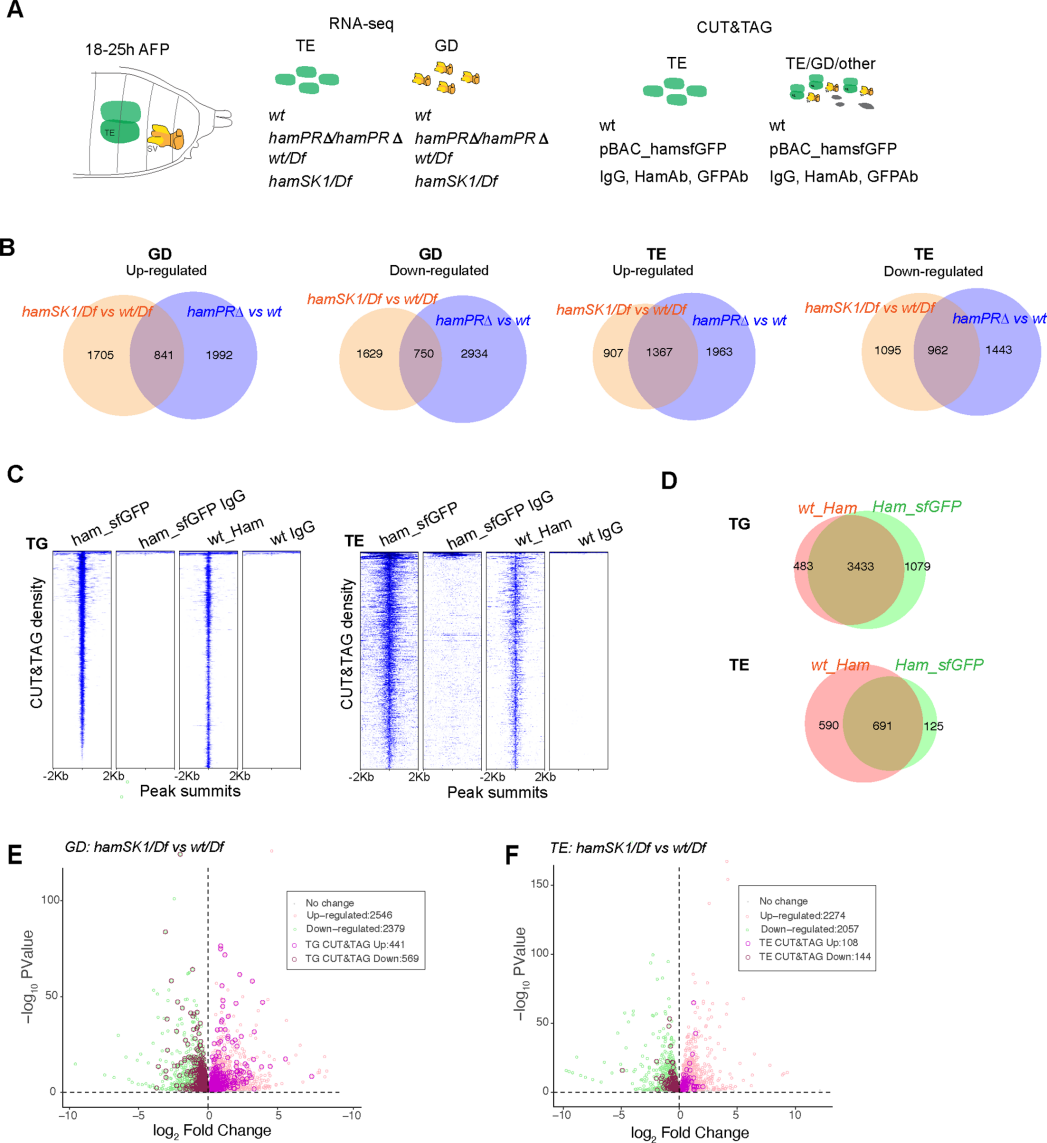
Identification of Ham-regulated genes in the developing TE and genital disc (GD). (**A**) Illustration of the sample types and antibodies used in the RNA-seq and CUT&TAG experiments. (**B**) Venn diagram of dysregulated genes between the two mutant conditions in the GD (left) and TE (right) samples. (**C**) Heatmaps of Ham CUT&TAG peak density and IgG control, ranked from high to low and centered at peak summits. (**D**) Venn diagrams of CUT&TAG peaks from the two types of samples. Volcano plots of dysregulated genes in GD (**E**) and TE (**F**) in the *hamSK1/Df* mutant, and the ones with at least one Ham CUT&TAG peak are highlighted.

To identify mechanisms that control epithelial fusion, we started to investigate Ham downstream target gene network. We performed RNA-seq experiments from dissected TE and SV tissues during the stage of 18–25 hr APF. We used two pairs of genotypes, including the wild-type (*wt*), and *ham^PRΔ^* homozygous and the *wt/Df* and *hamSK1/Df* trans-heterozygous (Figure 3A). The comparison between the first pair will determine dysregulated genes in the *ham^PRΔ^* hypomorphic condition, while the comparison between the second pair will identify genes that change expression between the null and *ham* heterozygous conditions.

**Figure 3—figure supplement 1.**
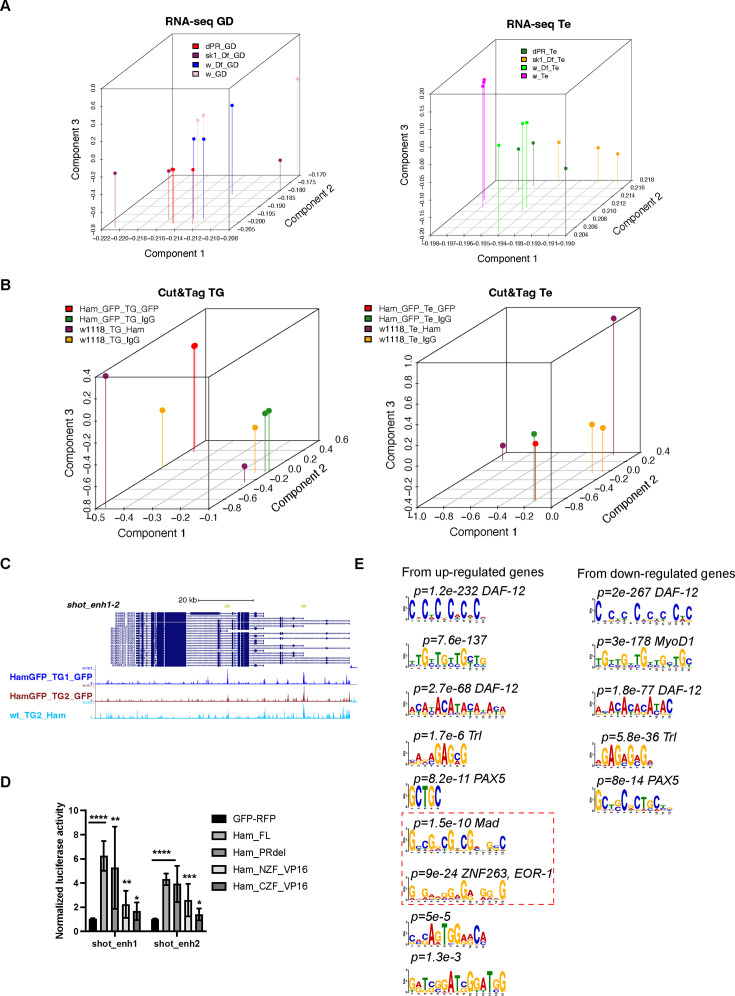
Ham regulatory activity and potential co-factors. (**A**) Principle component analysis of the RNA-seq datasets. (**B**) Principle component analysis of the Cut&Tag datasets. Note: some samples had poor sequencing depth, which were excluded from the PCA and later analyses. (**C**) The *shot* locus, showing the CUT&TAG tracks from the TE and GD (TG) mixed samples and the enhancer fragments in the luciferase assays. (**D**) Normalized luciferase activity to the empty reporter and then to the control vector that expresses GFP and RFP proteins. Each plot represents the average from three biological replicates with each biological replicate having four technical replicates. Statistical significance was calculated using unpaired *t*-test (****p < 0.0001; ***p < 0.001; **p < 0.01; *p < 0.05). (**E**) Significantly enriched motifs from Ham binding peaks in upregulated genes (left) and downregulated genes (right). The dashed rectangles highlight MAD and ZNF263 motifs. Figure 3—figure supplement 1—source data 1.Raw data of luciferase activity shown in Figure 3—figure supplement 1D.

The principal component analyses validated consistency between the three biological replicates (Figure 3—figure supplement 1A). To not exclude small gene expression changes (e.g. in the leading cells which are the minority within the whole tissue), we used a loose cutoff (false discovery rate [FDR] <10% and FC >1). We found more than 2000 up- and downregulated genes in the GD. In the mutant TE, there were similar numbers of dysregulated genes (Figure 3B). The genes that only changed expression in one condition could result from unspecific genetic background, differential dependence on the PR-containing isoforms or different sensitivity to *ham* heterozygous condition.

Expression of many dysregulated genes can be indirectly affected by Ham. We next intended to identify genes that are transcriptionally regulated by Ham. To this end, we determined Ham genomic binding sites using Cleavage Under Targets and Tagmentation assays (CUT&TAG) (Kaya-Okur et al., 2019) on finely dissected TE and grossly dissected abdominal tissues in which Ham is mainly expressed in the TE and SV epithelium (Figure 3A). We used a Ham antibody for wild-type tissues, a GFP antibody for tissues from the *pBAC_ham_SFGFP* transgene and IgG as a negative control in parallel for all the samples. Using the SEACR software, we identified peaks and intersections of peaks across the Ham and GFP antibody conditions (see Methods). We performed principal component analyses and found the GFP antibody gave more consistent peak calling from replicates (Figure 3—figure supplement 1B). For subsequent analyses, only the common 3433 and 691 peaks identified by the two antibodies (Ham and GFP) were included to ensure signal specificity (Figure 3C, D).

To determine Ham target genes, we intersected the RNA-seq data from the hamSK1/Df versus wt/Df comparison with the Cut&Tag data. Among the 2,546 upregulated genes in the GD, 441 (17.3%) contained Ham CUT&TAG peaks. Among the 2379 downregulated genes, 569 (23.9%) showed Ham binding (Figure 3E). In the TE, a lower fraction of dysregulated genes contains Ham CUT&TAG peaks, including 4.7% (108/2274) upregulated genes and 7% (144/2057) downregulated genes (Figure 3F), suggesting that dysregulated genes in the mutant TE are mainly indirectly controlled by Ham.

**Figure 4.**
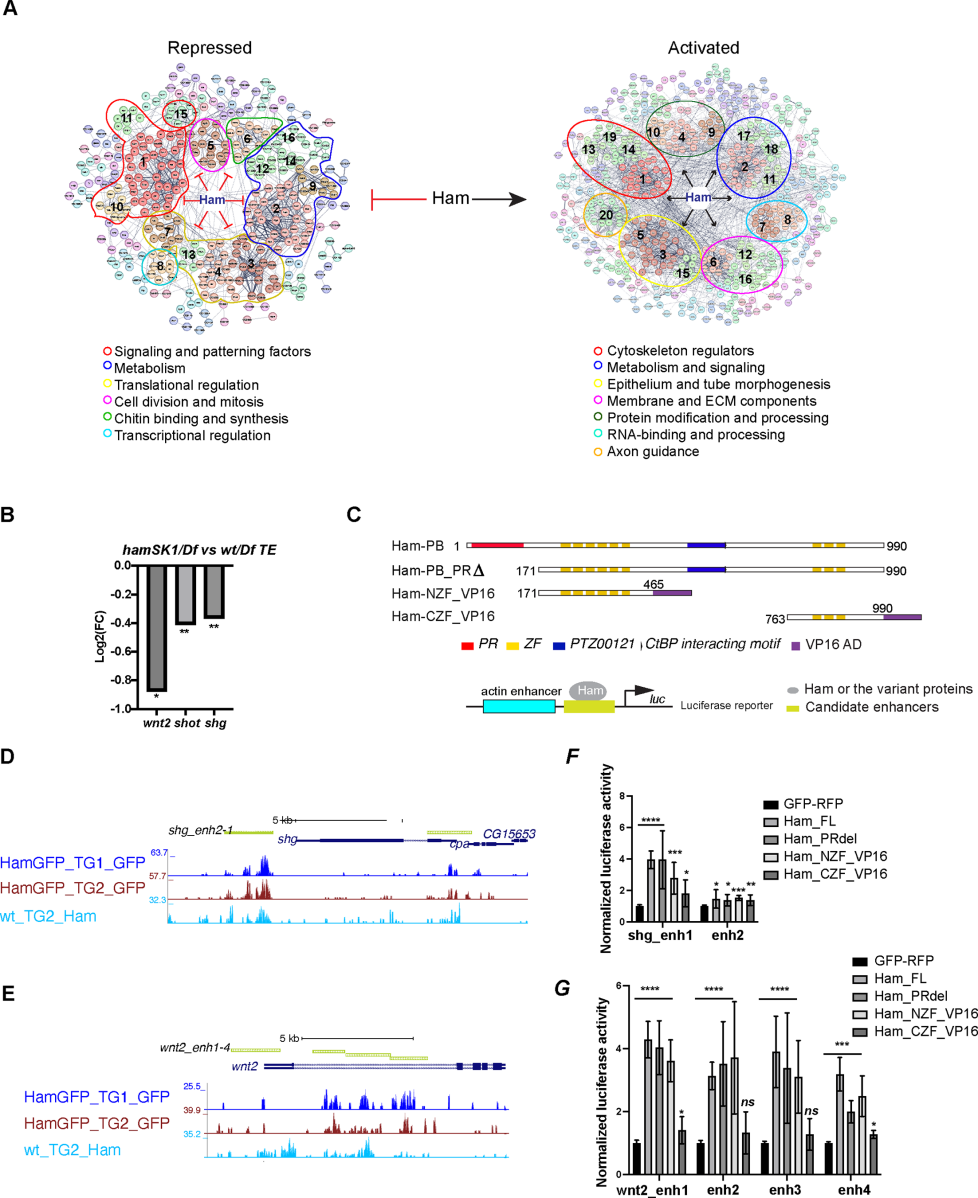
Ham activates expression of epithelial differentiation genes. (**A**) Ham gene network analysis from STRING. The position of MCL clusters were manually adjusted to place genes with similar function in close proximity. The functional categories were determined by gene function tools in STRING. (**B**) A bar plot of the log_2_ fold-change value from RNA-seq for *wnt2*, *shot*, and *shg*, showing downregulation of these genes in the *hamSK1/Df* mutant. (**C**) Schemes of the Ham_FL and other truncation proteins, and illustration of the luciferase reporter assay. (**D, E**) A genome browser snapshot of the *shg* and *wnt2* loci, showing the CUT&TAG tracks from the TE and GD (TG) mixed samples and the enhancer fragments in the luciferase assays. (**F, G**) Normalized luciferase activity to the empty reporter and then to the control vector that expresses GFP and RFP proteins. Each plot represents the average from three biological replicates with each biological replicate having four technical replicates. Statistical significance was calculated using unpaired *t*-test (****p < 0.0001; ***p < 0.001; **p < 0.01; *p < 0.05; ns, non-significant). Figure 4—source data 1.Raw data of luciferase activity shown in Figure 4F. Figure 4—source data 2.Raw data of luciferase activity shown in Figure 4G.

Both up- and downregulated genes contain Ham binding peaks, suggesting that Ham can repress and activate gene expression. To systematically explore the function of Ham-regulated genes, we performed gene network analysis using STRING (Szklarczyk et al., 2023). We focused on the genes bound by Ham and also dysregulated in the genital disc (GD) in the null condition *hamSK1*, considering that most of TE genes are only indirectly influenced in the *ham^SK1^* mutant. The SRING MSL clustering analysis generated 114 functional clusters from downregulated genes and 62 clusters from upregulated genes (Figure 4A, Supplementary file 1). Interestingly, many of downregulated genes clustered into several large groups including cytoskeleton regulators, metabolic factors, cell adhesion, and extracellular matrix proteins, suggesting that Ham activates genes to promote epithelial differentiation and remodeling. In contrast, upregulated genes are enriched for patterning factors, cell cycle genes, and transcriptional and translational regulators, suggesting that Ham suppresses early developmental processes. Notably, metabolic factors are enriched in both categories, but these factors belong to distinct functional groups. Thus, epithelial fusion requires complex and balanced metabolic processes. Together, this network analysis confirms that Ham positions on top of distinct molecular pathways to facilitate TE and SV assembly.

Ham was known for its repressive activity via interacting with the co-repressor CtBP and influencing histone modifications (Endo et al., 2012). Our finding of its activation function was unexpected. To validate the activation activity of Ham, we chose three downregulated target genes (Figure 4B), *dwnt2*, *shot*, and *shg*, for transcriptional reporter assays. *shg* encodes E-cad, while Shot is a member of the spectraplakin family of large cytoskeletal linker molecules simultaneously binding to actin and microtubules. The function of *wnt2* was reported in the developing RS development (Kozopas et al., 1998; Deshpande et al., 2016). We cloned four genomic fragments from the *wnt2* locus, two each from *shg* and *shot* into a luciferase construct, and co-transfected each reporter with individual plasmids expressing several variants of Ham. In addition to the Ham long isoform and a PR truncation version of Ham, we included the NZF and the CZF domains fused with the VP16 activation domain (NZF_VP16 and CZF_VP16) (Figure 4C). The two ZF domains of Ham are putative DNA binding domains, but it remained undetermined whether they help to recruit Ham to DNA. This experimental setting allows to assess which of the ZF domains is able to target Ham-regulated enhancers. The full-length, the PR truncation and the NZF_VP16 fusion all activated expression of the reporters driven by seven out of eight Ham-bound elements (Figure 4D–G, Figure 3—figure supplement 1C, D). By contrast, the CZF_VP16 had little activity on the reporters. This result confirms that Ham can directly activate target gene expression and that the NZF domain directly or indirectly recruits Ham to enhancers.

To further identify co-factors with which Ham interacts to regulate gene expression, we performed de novo motif discovery from Ham CUT&TAG peaks in mixed TE and GD tissues close to up- and downregulated genes in the *hamSK1* mutant using the MEME-ChIP software. This resulted in nine and five significantly enriched motifs from up- and downregulated gene loci, respectively (Figure 3—figure supplement 1E). While five motifs were shared between these two sets, the motifs for Dpp/BMP signaling factor MAD, the zinc-finger protein ZNF263 and two unknown factors were only enriched in the upregulated genes. The result implies that Ham may switch activation to repression activity when interacting with these co-factors in the context of RS development.

### Ham downstream effector genes are required for male RS fertility

Built upon our transcriptomic data, we next intended to identify genes crucial for the fusion process. We reasoned that as the majority of TE terminal epithelial cells are lost in both *ham* mutant alleles, the commonly downregulated genes in *ham^PRΔ^* and *ham^SK1^* TE (Figure 3B) may be expressed in the *wt* TE epithelial cells and can be functionally important. We thus focused on the top 241 common downregulated genes whose expression showed a fold change greater than 1.5, and the three validated Ham target genes: *shot*, *wnt2*, and *shg*. Using all available RNAi lines from the Bloomington stock center that cover 113 out of 244 candidate genes, we performed a UAS-RNAi screen with the *UAS-dcr2; hamRSGal4* driver for male fertility (see Methods).

**Figure 5.**
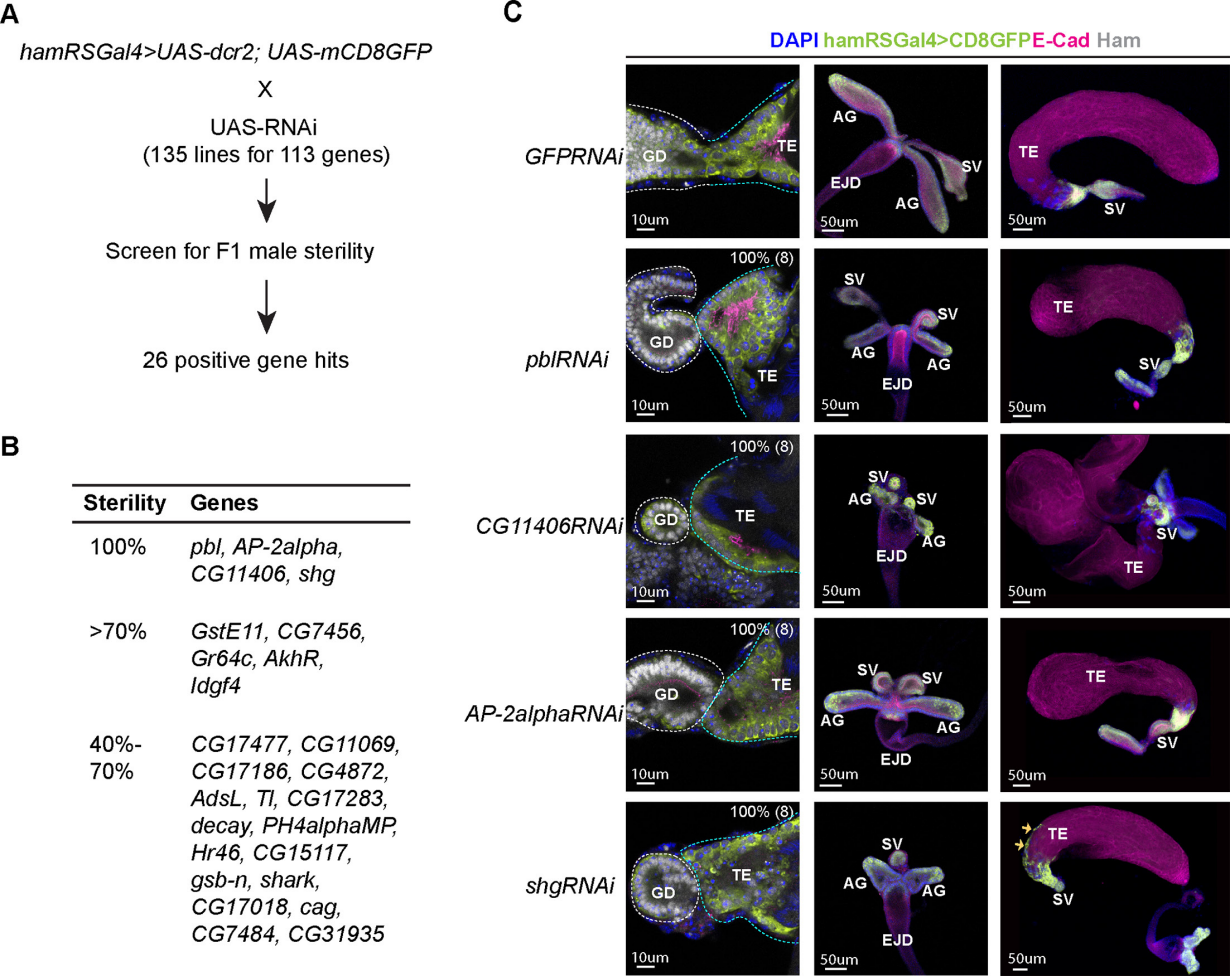
A candidate RNAi screen identified Ham-downstream effectors in reproductive system (RS) epithelial tissue fusion. (**A**) Scheme for the RNAi screen. (**B**) Summary of positive hits. The extent of sterility is determined by the number of progenies from the F1 male crossing to the wild-type female (see Methods). 100% means no progeny. >70%, the number of progenies being fewer than 30% of the number of progenies produced by the control cross (the same Gal4 driver × UAS-GFPRNAi). 40–70%, the number of progenies being between 30% and 60% of the number of progenies produced by the control. (**C**) Images of the control and gene-specific RNAi knockdown RS. The left panels show the fusion site stained with E-Cad in magenta, GFP in lime-green, Ham in gray and DAPI in blue, the middle panels are genital disc (GD), and the right ones are TE. The orange arrows indicate mobilized epithelial cells in the *shg* knockdown TE. Figure 5—source data 1.Results for a candidate RNAi screen shown in Figure 5A. Figure 5—source data 2.The extent of sterility for positive hits from the RNAi screen shown in Figure 5B.

The RNAi screen led to identification of 26 genes whose knockdown abolished or reduced male fertility (Supplementary file 2). Knocking down four of these genes, *shg*, *AP2-alpha*, *pebble* (*pbl*), and *CG11406*, resulted in complete sterility, indicating their importance for male fertility. We defined this category as 100% penetrance of sterility. Knockdown of other 22 genes reduced fertility to different extent (Figure 5A, B). Given that the *hamRSGal4* driver is highly expressed in the TE and SV epithelia, we expect highly effective knockdown occurs only in these epithelial cells. However, *hamRSGal4* also drives a weak expression in the myoblasts before they differentiated into myotubes (Figure 2—figure supplement 3B), which may result in a non-tissue autonomous effect when knocking down the candidate genes expressed in myoblasts. Furthermore, it cannot be excluded that knockdown in epithelial cells could indirectly influence myotube behavior, which could in turn perturb epithelial morphogenesis.

**Figure 5—figure supplement 1.**
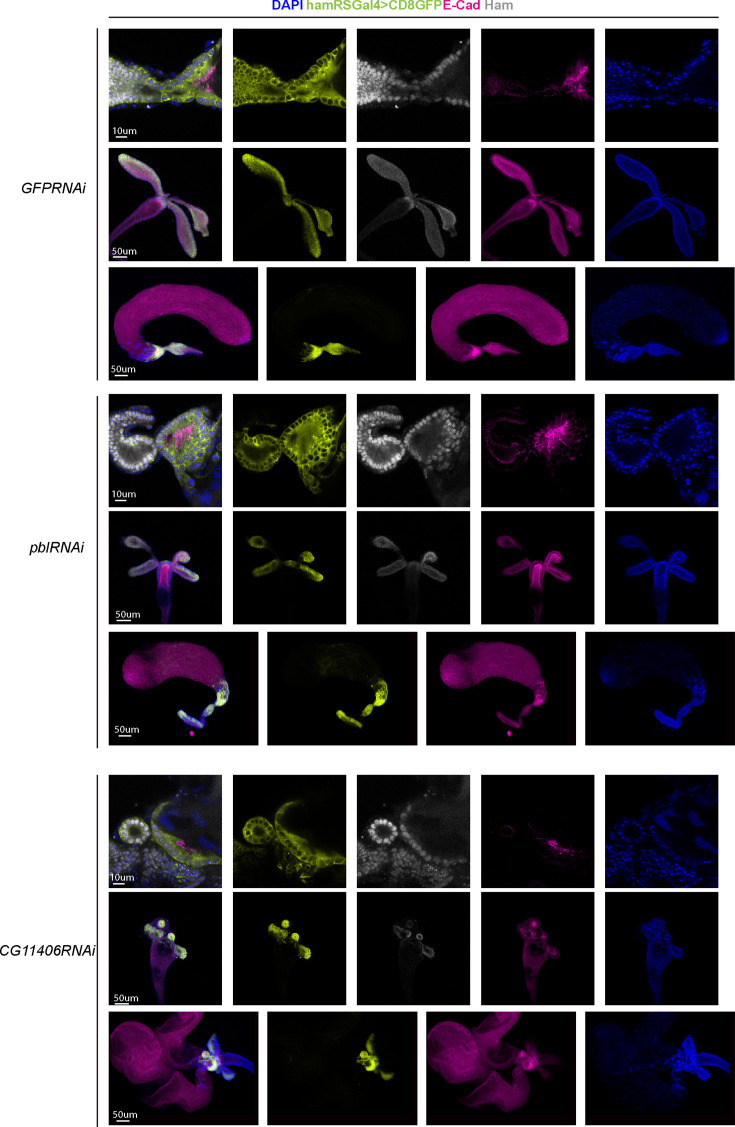
Split channel images for images in Figure 5.

**Figure 5—figure supplement 2.**
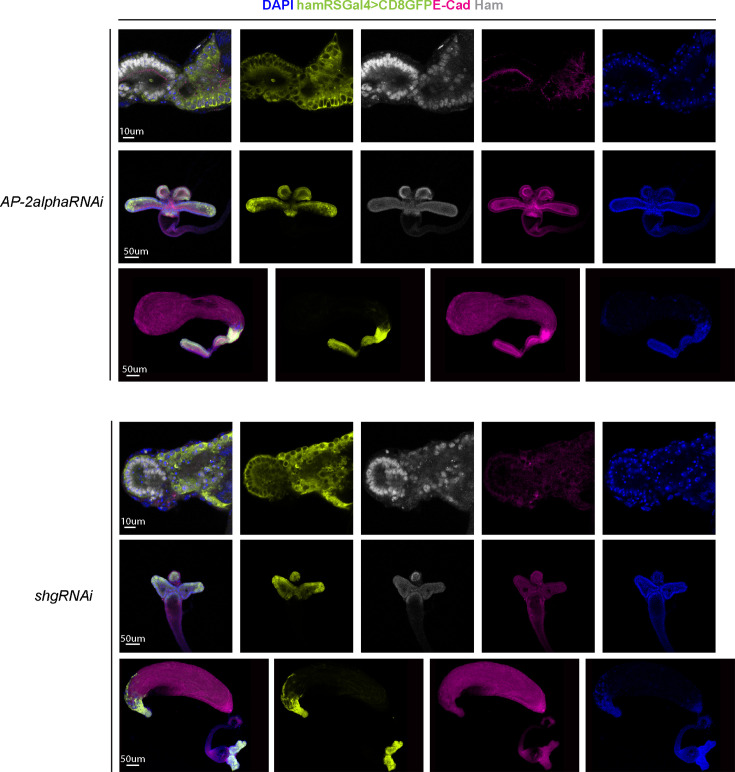
Split channel images for images in Figure 5.

Consistent with the gene network analysis (Figure 4A), these genes include cytoskeleton regulators (*pbl*, *shg*, and *shark*) (van Impel et al., 2009; Oda et al., 1994; Ferrante et al., 1995) and metabolism factors (*CG11406*, *Ph4alphaMP*, *GstE11*, *CG17477*, *CG11069*, *AdSL*, *CG17283*, *CG15117*, and *CG7484*). Other genes are known or predicted to be involved in intracellular membrane trafficking (*AP-2alpha*, *CG31935*, and *CG7456*) (González-Gaitán and Jäckle, 1997) and signaling (*wnt2*, *Toll*, *AkhR*, *Idgf4*, and *Hr46*) (Weber et al., 2003; Staubli et al., 2002; Kawamura et al., 1999; Lam et al., 1997).

We confirmed that knocking down *pbl*, *AP-2alpha*, *CG11406*, and *shg* led to failed TE and SV fusion and abnormal morphology at the pupal stage (Figure 5C, Figure 5—figure supplements 1 and 2). *pbl* encodes a Rho guanine nucleotide exchange factor, implicated in multiple processes involving cytoskeleton reorganization (van Impel et al., 2009; Prokopenko et al., 1999; Nakamura et al., 2017). The gene product of CG11406 is a predicted triglyceride lipase, cleaving triacylglycerol to produce diacylglycerol. AP-2alpha, a subunit in the Adaptor complex 2, is involved in intracellular trafficking and secretion (Lin et al., 2015). Remarkably, *shg* knockdown also led to aberrant mobilization of TE epithelial cells to the middle of the TE, while no such effect was found in the SV and AG. Downregulation of E-Cad is a key event during epithelial-to-mesenchymal transition, and E-Cad promotes cohesive cell migration of mesenchymal cells, but suppressing cell motility of epithelial cells (Campbell and Casanova, 2015; Chen et al., 1997). Our result suggests that the SV and the TE terminal epithelia have distinct dependence on E-Cad and that TE epithelial cells are prone to becoming motile when E-Cad is reduced. Together, this screen identified a collection of known and novel factors involved in epithelial tissue fusion, confirming that the fusion process requires coordinated activity of several classes of molecules.

### Ham downstream genes exhibit spatial–temporal expression dynamics in the male RS

During epithelial fusion, cells at the leading edge become specialized and express specific genes required for tissue interaction and remodeling (Bernascone et al., 2017; Chung and Andrew, 2008). Moreover, as we observed, the TE terminal and SV epithelia have distinct properties, suggesting that these two sets of epithelial cells may express distinct genes. We thus intended to assess spatial distribution of Ham downstream genes. We conducted a high-resolution multiplexed RNA in situ hybridization assay known as SCRINSHOT (single-cell RNA in situ hybridization on tissues) (Sountoulidis et al., 2020) to visualize expression of candidate genes. These genes include *wnt2*, *shg*, and *shot* as these genes were either known for male RS development (*wnt2*, Kozopas et al., 1998) or epithelial tissue fusion (*shg*, Lee et al., 2003 and *shot*, Lee and Kolodziej, 2002), and the positive genes from the RNAi screen, along with the receptor genes in Wnt2 signaling (*fz*, *fz2*, *fz3*, and *fz4*) and ligand genes in Tl-signaling (*spz*, *spz4*, *spz5*, and *spz6*) (Supplementary file 3). Some of these Wnt2 and Tl signaling genes displayed dysregulation in at least one of the *ham* mutant alleles according RNA-seq data (Figure 6—figure supplement 1A, B). Other receptor genes in Wnt signaling and ligand genes in Tl signaling were omitted due to low read numbers in the RNA-seq data, indicating negligible or minimal expression in the TE and GD.

**Figure 6.**
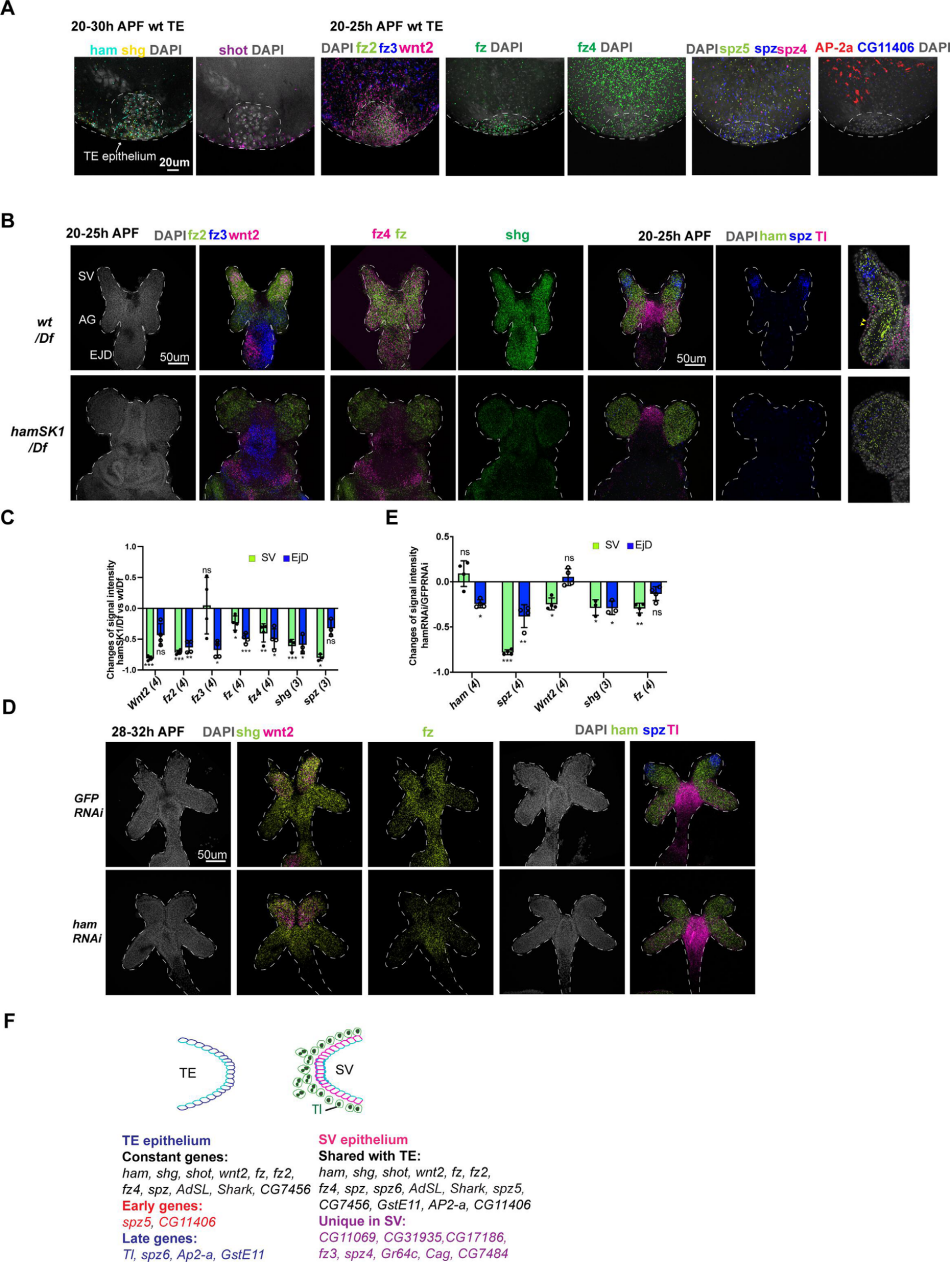
Spatial–temporal expression dynamics of Ham downstream genes. (**A**) Images from multiplexed in situ hybridization (the SCRINSHOT method), showing mRNA signal of the indicated genes in *wt* TE samples. DNA is marked by DAPI in gray. (**B**) Images from SCRINSHOT, showing mRNA signals of indicated genes in *wt/Df* and *hamSK1/Df* mutant GD. The last panel is an enlarged view from the sample with *Tl*, *ham*, and *spz* probes. The yellow arrowheads indicate myoblast cells with Tl signal. (**C**) Quantification of signal intensity for genes shown in B in two areas, the SV and EjD. Statistical significance was calculated using unpaired *t*-test (***p < 0.001; **p < 0.01; *p < 0.05; ns, non-significant). (**D**) Images from SCRINSHOT, showing mRNA signals of indicated genes in *GFP* and *ham RNAi* GD samples. (**E**) Quantification of signal intensity for genes shown in D in two areas, the SV and EjD. Statistical significance was calculated using unpaired *t*-test (***p < 0.001; **p < 0.01; *p < 0.05; ns, non-significant). (**F**) A summary of genes expressed in the TE terminal and SV epithelium. Highlighted genes show temporal or spatial dynamic expression in the two tissues. Figure 6—source data 1.Quantification of SCRINSHOT signal intensity changes for indicated genes between *hamSK1/Df* and *wt/Df* in the areas of SV and EjD, as shown in Figure 6C. Figure 6—source data 2.Quantification of SCRINSHOT signal intensity changes for indicated genes between *hamRNAi* and *GFPRNAi* in the areas of SV and EjD, as shown in Figure 6E.

Each SCRINSHOT reaction took place within a mini-chamber, accommodating pairs of control and *ham* mutant tissues, or GFP KD control and ham RNAi KD samples (refer to Methods for details of probe design and experimental procedures). This setup was designed to minimize technical variability between the genotypes. mRNAs are visualized as fluorescent dots in SCRINSHOT images, with each dot representing a single mRNA molecule. However, due to the densely packed nature of cells in the TE and SV tissues, quantifying mRNA molecules at a single-cell resolution is challenging. Therefore, we opted to quantify fluorescent signal intensity within defined areas (see Methods). In total, 37 genes were tested, including *ham* itself (Supplementary file 3). Probes for seven genes gave either no or low signal in the TE or GD, likely attributed to poor probe quality, given their RNA-seq read numbers not being low. Resembling Ham protein expression (Figure 1F), *ham* mRNAs were detected in the TE terminal epithelium (Figure 6A, Figure 6—figure supplement 2A), confirming the specificity of the SCRINSHOT method. Similarly, *wnt2*, *shg*, and *shot* all exhibited high expression in the TE terminal epithelium (Figure 6A, Figure 6—figure supplement 2A).

**Figure 6—figure supplement 1.**
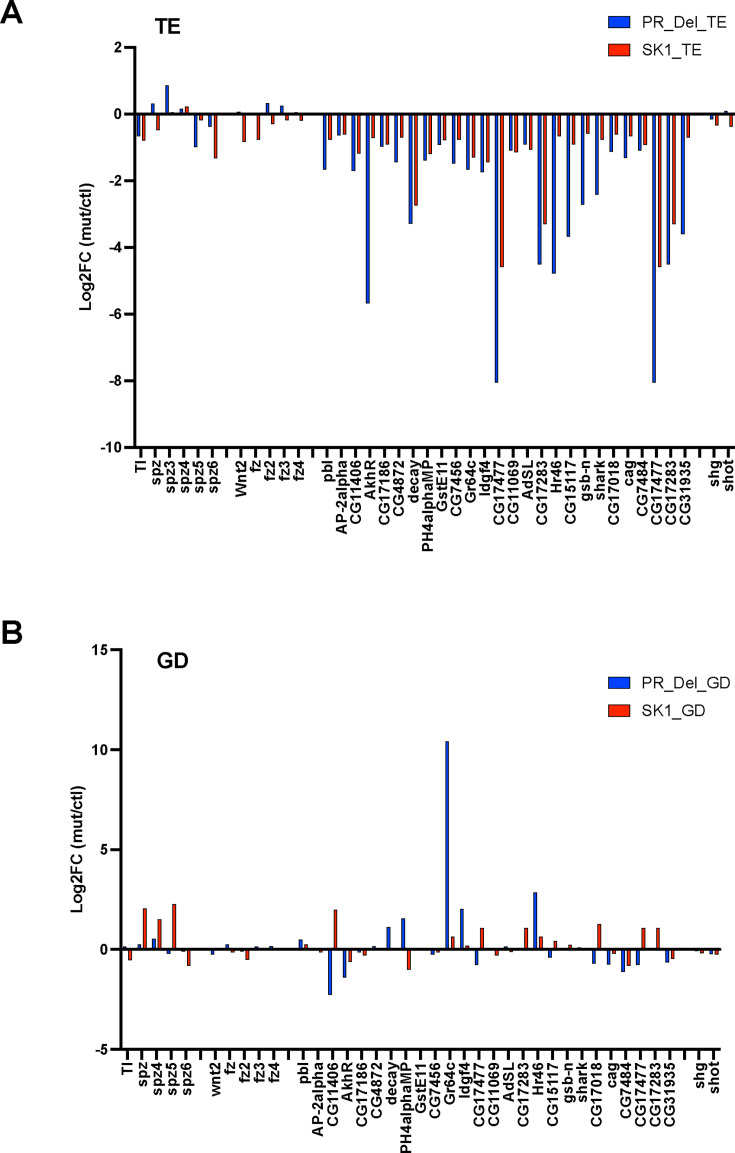
Expression of genes tested by SCRINSHOT in RNA-seq data. (**A**) Log_2_FC (hamSK1/Df versus wt/Df and *hamPRΔ* versus wt) in TE. (**B**) Log_2_FC (hamSK1/Df versus wt/Df and *hamPRΔ* versus wt) in genital disc (GD).

**Figure 6—figure supplement 2.**
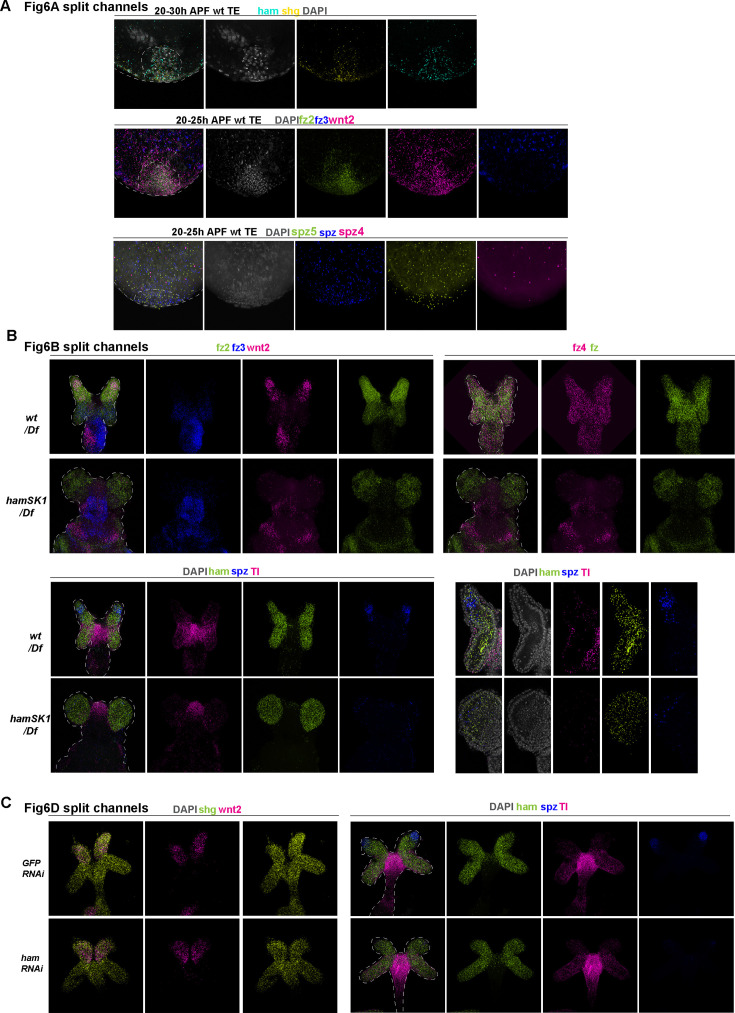
Split channel images for images in Figure 6.

**Figure 6—figure supplement 3.**
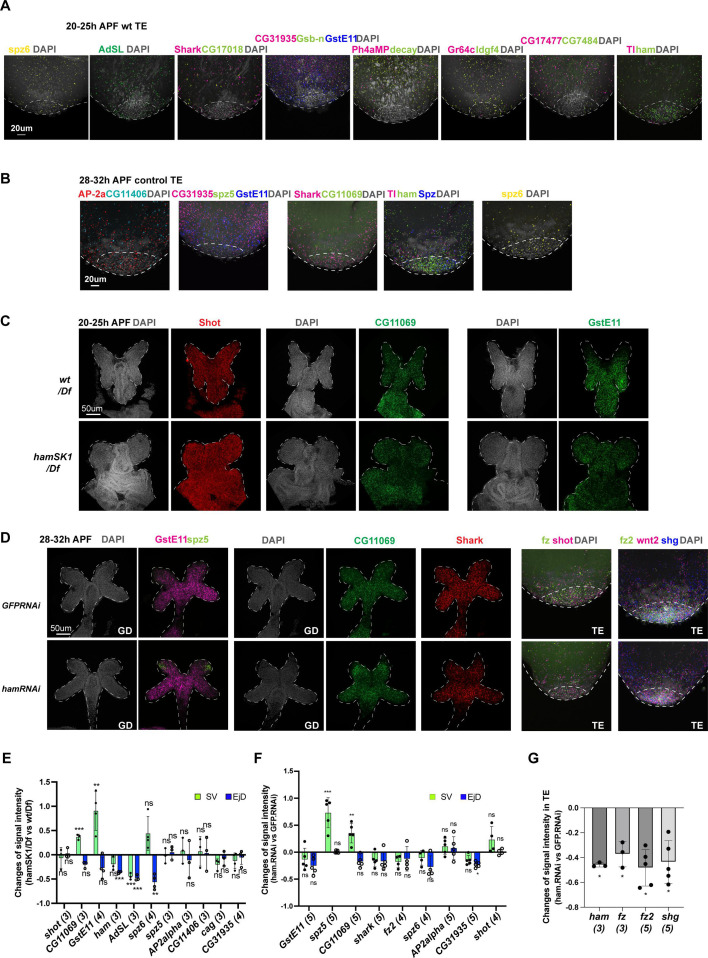
Additional genes tested in SCRINSHOT. (**A**) Images from SCRINSHOT, showing mRNA signal of the indicated genes in *wt* TE samples. DNA is marked by DAPI in gray. (**B**) Images from SCRINSHOT, showing mRNA signal of the indicated genes in *wt* TE samples at a later pupal stage (28–32 APF). DNA is marked by DAPI in gray. (**C**) Images from SCRINSHOT, showing mRNA signals of indicated genes in *wt/Df* and *hamSK1/Df* mutant GDs. (**D**) Images from SCRINSHOT, showing mRNA signals of indicated genes in *GFP* and *ham RNAi* GD samples. (**E, F**) Quantification of signal intensity for genes shown in C and D in two areas, the SV and EjD. (**G**) Quantification of signal intensity for genes that changed expression in ham RNAi TE. Statistical significance was calculated using unpaired *t*-test (***p < 0.001; **p < 0.01; *p < 0.05; ns, non-significant). Figure 6—figure supplement 3—source data 1.Quantification of SCRINSHOT signal intensity changes for indicated genes between *hamSK1/Df* and *wt/Df* in the areas of SV and EjD, as shown in Figure 6—figure supplement 3E. Figure 6—figure supplement 3—source data 2.Quantification of SCRINSHOT signal intensity changes for indicated genes between *hamRNAi* and *GFPRNAi* in the areas of SV and EjD, as shown in Figure 6—figure supplement 3F. Figure 6—figure supplement 3—source data 3.Quantification of signal intensity for genes that changed expression in *ham RNAi* TE shown in Figure 6—figure supplement 3G.

**Figure 6—figure supplement 4.**
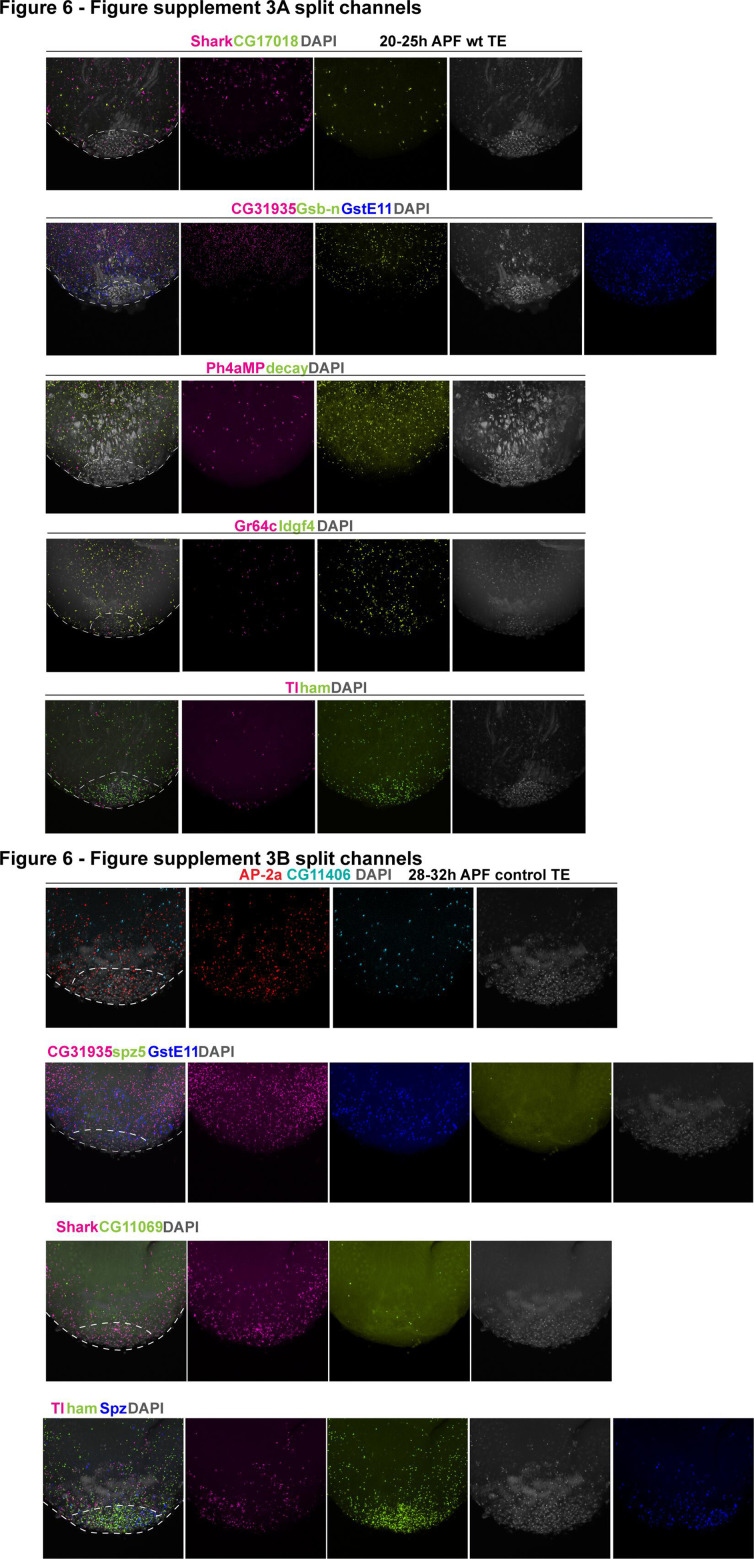
Split channel images for images in Figure 6—figure supplement 3.

**Figure 6—figure supplement 5.**
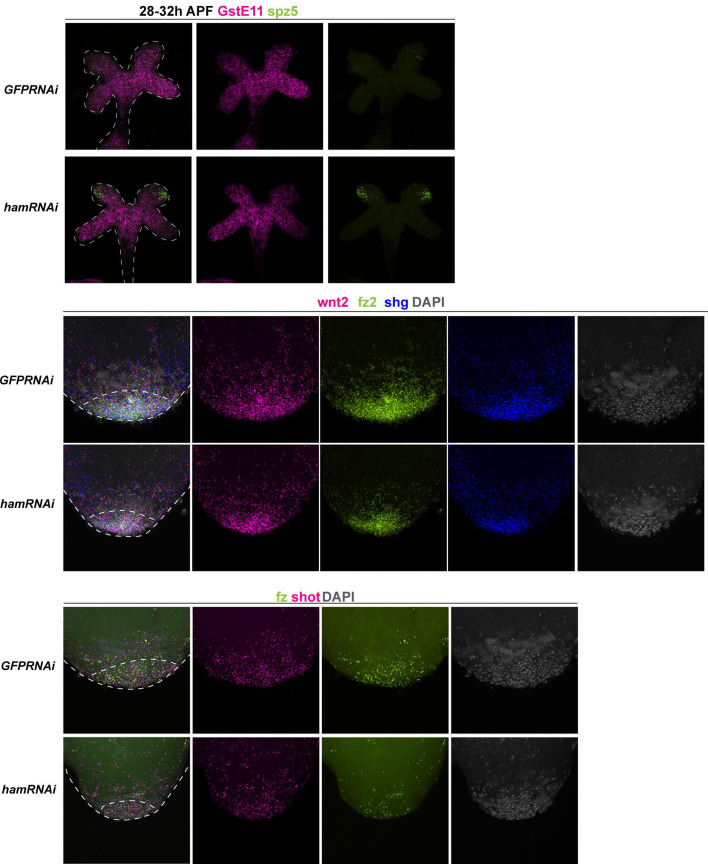
Additional split channel images for the images in Figure 6—figure supplement 3.

In total, 25 genes exhibited detectable SCRINSHOT signal in the wt SV and AG, while 29 displayed signals in the wt TE (Supplementary file 3). Within the TE, 17 out of 29 were specifically localized in the TE terminal epithelial cells, with the remaining 12 detected only in other TE cells (Figure 6A, Figure 6—figure supplements 2–5, Supplementary file 3).

Subsequently, our analysis focused on the genes expressed in the TE terminal and SV epithelia. While 17 genes exhibited expression in both epithelial ends (Figure 6A, B, Figure 6—figure supplement 2A, B), 8 were exclusively expressed in SV epithelial cells (Figure 6F, Supplementary file 3), underscoring the heterotypic nature of the TE and SV epitheliums. Notably, certain genes (CG11406, *AP-2alpha*, *GstE11*, *Tl*, *spz5*, and *spz6*) demonstrated temporal dynamic expression in the TE epithelium: spz5 and CG11406 are present during the 20–25 hr APF timeframe, prior to fusion, but gone during the 28–32 hr APF period when the TE and SV are undergoing fusion; *AP-2alpha*, *GstE11*, *Tl*, and *spz6* showed the opposite trend, being expressed only later (Figure 6, Figure 6—figure supplements 2–4).

In the GD, while the majority of genes exhibited ubiquitous expression, *wnt2* and *spz* were predominantly expressed in the leading cells within the SV, contrasting with their expression in the remaining GD (Figure 6B). Additionally, the expression patterns of *fz2*, *fz3*, and *Tl* displayed spatial specificity: fz2 exhibited higher expression levels in the SV compared to the AG, *fz3* demonstrated the opposite pattern with higher expression in the AG, and *Tl* was primarily expressed in the myoblasts (Figure 6B). These findings imply that Wnt2 and Tl signaling may have cell-type-specific roles in the fusion process. Thus, SCRINSHOT mapped 30 candidate genes in the developing GD and TE, with 29 of them display spatial or temporal dynamics.

### Wnt2 and Tl signaling pathways are key mediators of Ham function in RS tissue assembly

Next, we aimed to identify genes exhibiting altered expression in *ham* mutant SV and TE. As *hamSK1* mutant TE lacks terminal epithelial cells, we excluded TE samples and only quantified SCRINSHOT signal levels in the developing SV between *wt* and the *hamSK1* mutant. In contrast, *ham* RNAi knockdown resulted in fusion defects but only mildly impacted SV morphology and TE terminal epithelial cell number (Figure 2G, H). These samples allow to assess gene expression alterations in both the SV and TE epithelia. *ham* fluorescent intensity appeared comparable between *wt* and mutant samples (Figure 6B, D, E, Figure 6—figure supplements 2–5), suggesting that *ham* mutant RNAs, containing the CRISPR-generated indels, remain stable at this stage. In the *hamSK1* mutant SV, seven genes became downregulated, while two exhibited upregulation. The downregulated genes were four Wnt2 pathway genes (*wnt2*, *fz*, *fz2*, and *fz4*), one Tl pathway gene (*spz*), and *shg* (Figure 6B, C, Figure 6—figure supplement 2). Notably, *wnt2*, *fz*, *spz*, and *shg* also displayed downregulation in *ham* RNAi SV (Figure 6D, E, Figure 6—figure supplement 2), suggesting that these genes and their associated signaling pathways may be directly involved in the fusion process downstream of Ham. The upregulated genes in *hamSK1* mutant SV include *GstE11* and *CG11069* (Figure 6—figure supplement 3C, E), which exhibited low expression in the leading cells of wt SV epithelium, but increased expression under the *hamSK1* condition. In *ham* RNAi knockdown animals, *CG11069* was again upregulated, while *GstE11* did not change expression. Instead, another ligand gene in Tl signaling (*spz5*) displayed increased expression (Figure 6—figure supplement 3D, F). On the TE side, only four genes showed expression alteration in *ham* RNAi animals, including *ham* itself, *shg*, *fz*, and *fz2* (Figure 6—figure supplement 3G), implicating functional requirement of these genes in both TE and SV epithelia.

The observation that knocking down *Tl* resulted in decreased male fertility (Figure 5B, Supplementary file 2), coupled with the expression of multiple *spz* ligand genes in the developing TE and SV, suggests that the Tl signaling pathway might be required for assembling the TE and SV. To investigate this possibility, we examined male flies with *spz* knockdown driven by *hamRSGal4*. These animals produced a comparable number of progenies to control animals. The lack of phenotype in this condition could be attributed to inefficient knockdown or genetic compensation by another *spz* gene, like *spz5* whose expression increased in *ham* RNAi animals when *spz* expression was reduced. In addition to Tl, two other Tl related genes (Toll-7 and Toll-9) exhibited moderate number of reads in the RNA-seq data. We then tested male fertility in *Toll-7* and *Toll-9* knockdown animals. *Toll-9* knockdown led to a drastic reduction in progeny numbers compared to the RNAi control (Figure 7—figure supplement 1A). Consistent with the sterility, some of the *Toll-9* knockdown males displayed abnormal TE shape (Figure 7—figure supplement 1B). Remarkably the majority of Toll-9 knockdown animals showed empty SV without sperms (Figure 7—figure supplement 1C), suggesting failed connection between the TE and SV. Together, these results pinpoint an essential role of Tl signaling in male RS development.

**Figure 7.**
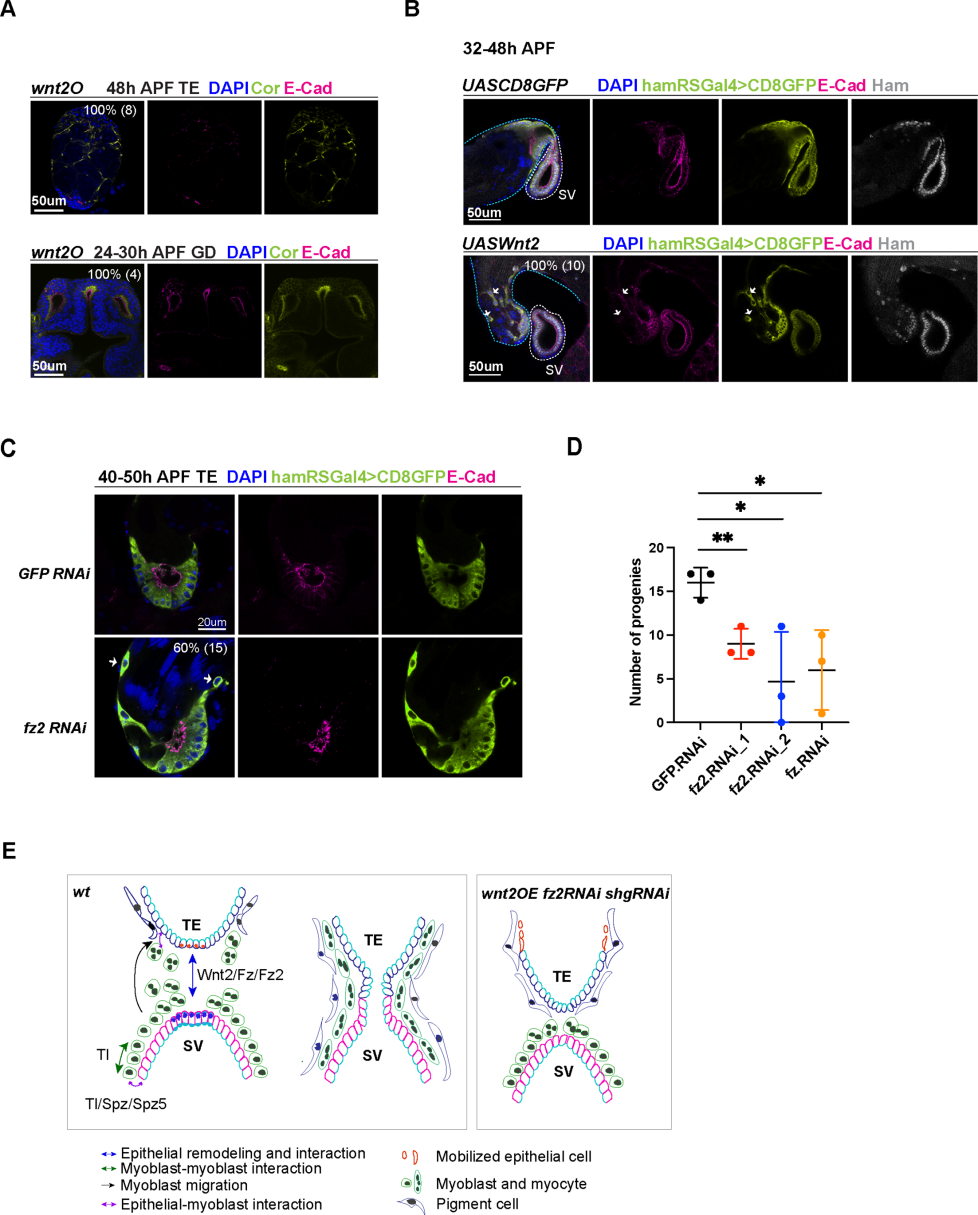
Wnt2 and its ligand genes are required for male fertility and reproductive system (RS) development. (**A**) Images of *wnt2o* mutant TE and GD, showing disrupted morphology in these tissues. (**B**) Images of the TE and SV fusion site from the control and *wnt2* overexpression animals. The epithelial cells of the TE and SV are labeled with hamRSGal4 driving UAS-mCD8GFP in lime-green and E-Cad in magenta and Ham in gray. The white arrows indicate mobilized GFP positive cells in the middle of the TE, and these cells have a lower level of E-Cad. The blue and white dashed lines highlight the TE and SV, respectively. (**C**) Images of the TE and SV fusion site from the control and *fz2* RNAi animals. The white arrows indicate mobilized GFP positive cells in the middle of the TE, and these cells have a lower level of E-Cad. (**D**) Graphs of progeny number from male fertility tests for *fz* and *fz2* RNAi lines in comparison to GFP RNAi. n = 3. Statistical significance was calculated using unpaired t-test (**, p< 0.01; *, p<0.05). (**E**) Illustration of cell–cell interactions in the developing RS, and a speculated model for Tl and Wnt2 signaling functions. Wnt2 signaling may be involved in epithelial cell interaction between the two ends. Tl signaling and Tl may be involved in the interaction between epithelial cells and myoblasts/myotubes. In three conditions (*shgRNAi*, *wnt2OE*, and *fz2RNAi*), some TE terminal epithelial cells (illustrated in orange) move up to the distal end of the testis. Figure 7—source data 1.Number of progenies from male fertility tests for *fz* and *fz2*.*RNAi* lines in comparison to *GFP.RNAi* shown in Figure 7D.

To validate the role of Wnt2 signaling in RS development, we investigated four genetic conditions, aimed at modulating its level and activity: *wnt2^O^* (a *wnt2* null allele), *wnt2* overexpression driven by *hamRSGal4*, *fz*, and *fz2* RNAi knockdown driven by *hamRSGal4*. The *wnt2^O^* mutant TE and GD never connect, suggesting that Wnt2 is essential for the fusion process. The *wnt2^O^* mutant TE lacks the pigment cell and myocyte layers (Figure 7A), consistent with its reported function in these two cell types (Rothenbusch-Fender et al., 2017; Kozopas et al., 1998). The *wnt2^O^* mutant GD failed to form elongated SV and AG, suggesting that Wnt2 is required for GD patterning and differentiation. Moreover, overexpression of *wnt2* also resulted in fusion defects and mis-localization of TE epithelial cells (Figure 7B), a phenotype reminiscent of *shg* knockdown animals. Indeed, *wnt2*-overexpressing cells displayed reduced levels of E-Cad, suggesting that Wnt2 signaling might balance E-Cad levels to maintain TE epithelial cell properties. Consistently, *fz2* knockdown animals exhibited comparable phenotypes, including mobilized TE epithelial cells and failed tissue fusion (Figure 7C). *fz2* and *fz* knockdown also decreased male fertility (Figure 7D). Collectively, these results suggest the importance of optimal Wnt2 signaling levels in male RS development and epithelial tissue fusion.

**Figure 7—figure supplement 1.**
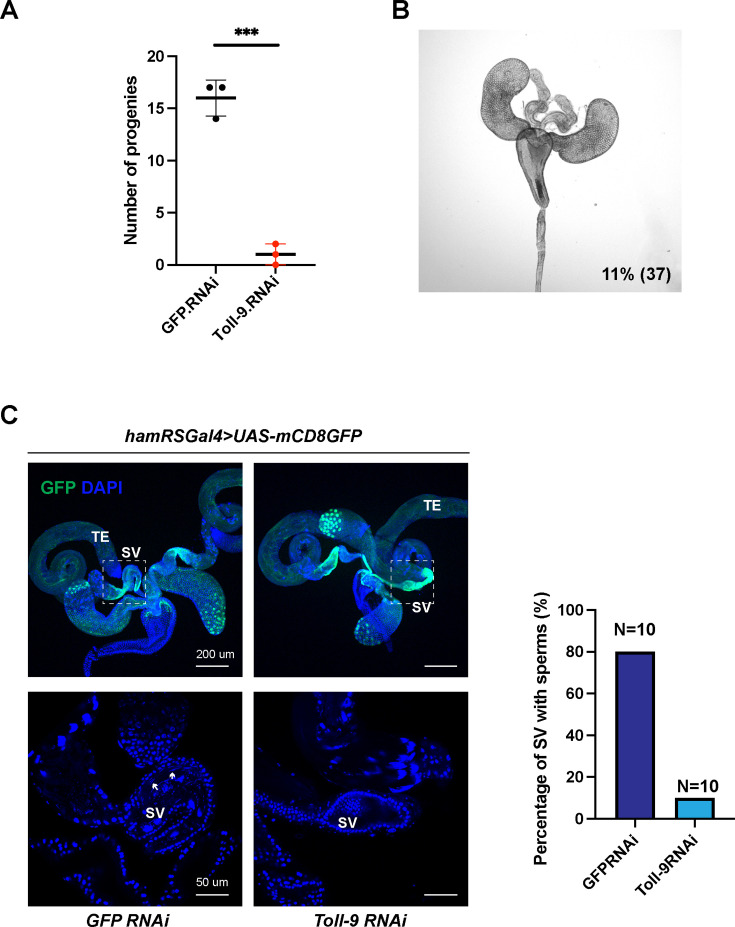
Male fertility test for Toll-9. (**A**) Graphs of progeny number from male fertility test for hamRSGal4 driving *Toll-9* RNAi in comparison to GFP RNAi. The number of tested males = 3. Statistical significance was calculated using unpaired t-test (***, p<0.001). (**B**) White-field image of Toll-9 RNAi adult male reproductive system (RS), showing morphological defects of the TE and the phenotype penetrance (11% of 37 animals). (**C**) Images of adult RS from control GFP RNAi andToll-9 RNAi males. hamRSGal4 >UAS-mCD8GFP is used to label the testis terminal epithelium and the SV, and DAPI stains DNA. The panels below are higher magnification views on the selected SV area, highlighted with white dashed squares. White arrows indicate sperms in the control SV, which are absent from the Toll-RNAi sample. The bar graph shows the percentage of SV with sperms in GFP and Toll-9 RNAi animals (*N* = 10). Figure 7—figure supplement 1—source data 1.Number of progenies from male fertility test for hamRSGal4 driving *Toll-9 RNAi* in comparison to *GFP RNAi* shown in Figure 7—figure supplement 1A.

## Discussion

In this study, we demonstrate that the transcription factor Ham plays a crucial role in controlling epithelial fusion during *Drosophila* RS development. Early in embryonic development, Ham determines the cell fate of msSGPs, which are progenitor cells for TE terminal epithelial cells. Later on, Ham directly regulates the differentiation of epithelial cells in both the TE and SV by orchestrating a gene expression program in epithelial cells. While our primary focus is on male RS, we found that Ham is also expressed in cells at the fusion site of the oviduct and ovary. Mutant females lacking functional Ham exhibit a non-functional oviduct epithelial tube, suggesting that Ham likely employs a similar mechanism to control epithelium fusion in females.

By examining *ham* mutant phenotypes and its downstream target genes, we identified several classes of novel factors involved in fusion process of SV and TE. These genes, directly or indirectly controlled by Ham, encompass a diverse array of molecular functions. This discovery provides a comprehensive gene network for future investigation of epithelial fusion processes. The cell-type- and tissue-specific spatial distribution of these genes suggest that they may regulate various aspects of fusion, including cell migration, intercellular communication between myoblasts/myotubes and epithelial cells, specification of the leading cells, and other steps in the fusion process. For instance, Tl and its related receptors is known to mediate cell adhesions (Umetsu, 2022). We can speculate that Tl signaling and the molecule itself promote interaction between myoblasts and myoblasts and epithelial cells (Figure 7E), based on high expression of Tl in myoblasts and the presence of multiple Spz ligands in the TE and SV epithelial cells. Moreover, *Wnt2*, *fz*, and *fz2* are highly expressed in the leading cells of TE and SV epithelia, and also required for TE and SV connection, suggesting that Wnt2 signaling mediates epithelial cell communication, remodeling, and fusion.

**Figure 8.**
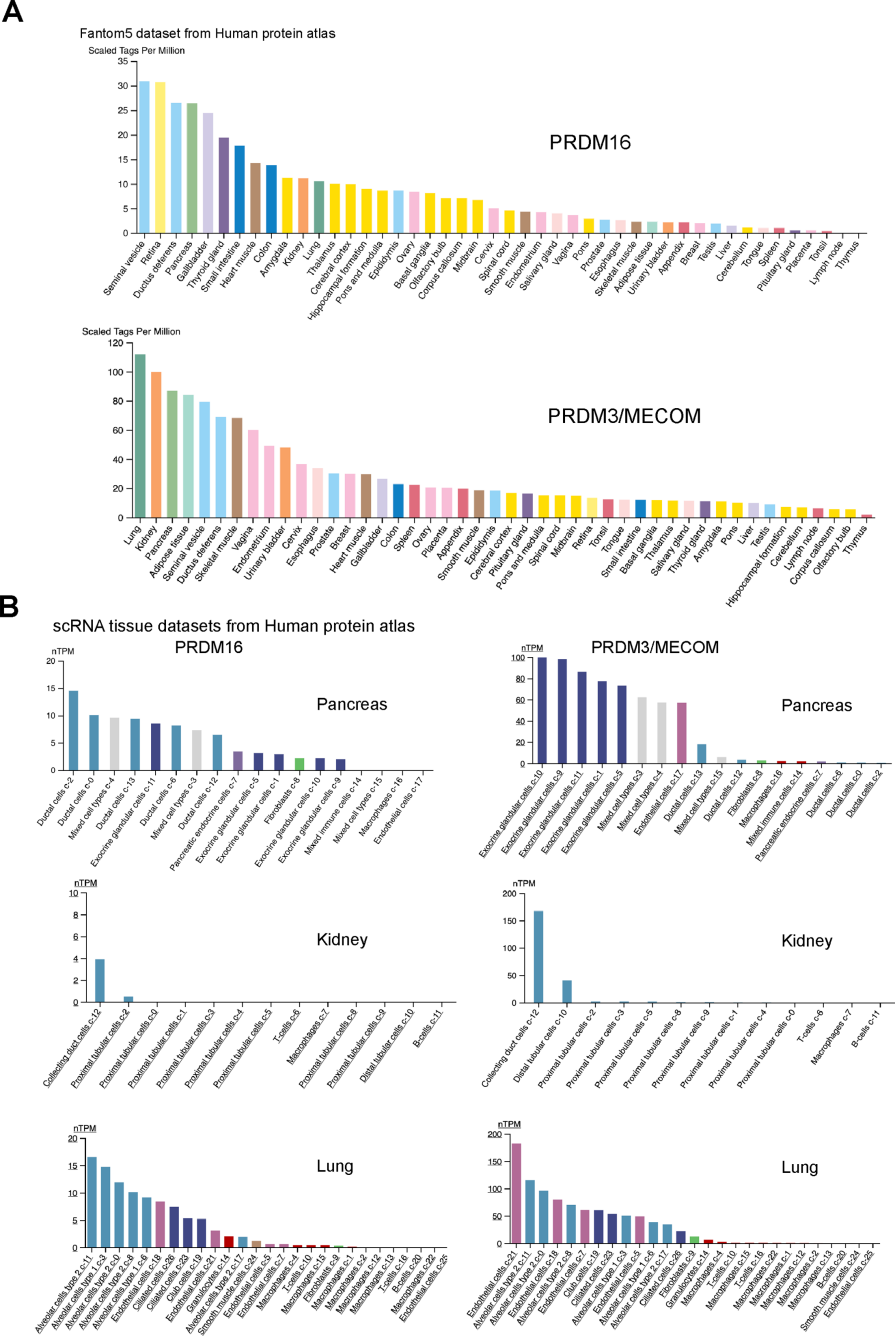
PRDM16 and MECOM/PRDM3 expression in human tissues. (**A**) RNA expression data across human tissues through Cap analysis of gene expression (Fantom5 dataset from the Human Protein Atlas). (**B**) Expression in single-cell RNA-seq clusters in the indicated human tissues (from the Human Protein Atlas).

Despite being discovered more than 20 years ago (Moore et al., 2002), research on the function of Ham function has primarily focused on its roles in the nervous system. Our findings highlight a crucial function of Ham in epithelial tissues. Apart from the RS, we observed high expression of the Ham protein in other epithelial tissues including the Malpighian tubes (akin to the kidney in insects) and the intestine (data not shown). Furthermore, orthologs of Ham, PRDM16, and PRDM3/MECOM, exhibit high expression in epithelial tubular structures, such as lung, pancreas, kidney, urinary bladder, and gastrointestinal tract and lung, according to observations in the Human Protein Atlas (Figure 8, PRDM16, MECOM) (Karlsson et al., 2021). Notably, SV and ductus deferens, the equivalent structure to fly SV, are the top one and three tissues with the highest PRDM16 expression (Figure 8A). This suggests a potential role for these proteins in controlling epithelial integrity and fusion in mammalian organogenesis. Indeed, we observed that the pancreas duct volume in mouse embryos lacking *Prdm16* is significantly reduced (data not shown). Although the exact cellular changes and the underlying molecular mechanisms in this context await further investigation, this phenotype underscores a crucial function of *Prdm16* in pancreatic epithelial duct formation.

We show that compared to Ham function in the nervous system, the PR-containing isoforms are indispensable for RS epithelial fusion and differentiation, and that Ham can directly activate epithelial gene expression. It remains to be determined whether Ham has distinct molecular functions or it regulates a subset of common molecular pathways in these two contexts. Dendrite morphogenesis involves outgrowth and branching, somewhat analogous to epithelial tube growth and branching. For instance, molecular mechanisms regulating polarized protein trafficking are crucial for both dendrite and epithelial tube branching (Corty et al., 2009). Additionally, several transcription factors like Sequoia (Araújo and Casanova, 2011; Brenman et al., 2001), Tramtrack (Araújo et al., 2007; Li et al., 1997), and Cut (Pitsouli and Perrimon, 2013; Grueber et al., 2003) play roles in both dendrite morphogenesis and tracheal tube fusion. These examples suggest that there might be more unrecognized common mechanisms between dendrite and epithelial morphogenesis. It is of interest to test whether the effectors we identified in the RS epithelium are involved in dendrite morphogenesis in the nervous system.

We observed a unique aspect of epithelial fusion in the *Drosophila* RS. Unlike many epithelial fusion processes where two homologous ends merge, such as those in mammalian neural tube closure and *Drosophila* tracheal formation, etc. (Bernascone et al., 2017; Jacinto et al., 2001; Pai et al., 2012), the two epithelial ends in the *Drosophila* RS are heterotypic. Specifically, the TE terminal epithelial cells express higher levels of E-Cad and lower levels of Cor compared to SV epithelial cells. In response to the reduction of E-Cad or ectopic expression of *wnt2*, TE terminal epithelial cells become motile, resembling a process akin to epithelial-to-mesenchymal transition. This suggests that TE terminal epithelial cells may be more invasive during epithelial fusion. On the other hand, SV epithelial cells, despite originating from mesenchymal cells, exhibit typical epithelial cell characteristics, including regular Cor and E-Cad expression. Notably, many genes are expressed exclusively in the SV epithelium, distinguishing it from the TE epithelium. Heterotypic epithelial fusion occurs in many developmental processes, such as digestive system formation, vertebrate optic fissure closure, and mammalian urogenital development. Failure of epithelial fusion can lead to a wide variety of developmental diseases such as oesophageal atresia, spina bifida, ocular coloboma, congenital abnormalities of the kidney, urinary tract, and persistent cloaca. Studying the basic processes and key players of normal epithelial tissue fusion in an in vivo context will help to understand the pathogenesis of these diseases.

## Materials and methods

### Fly strain culturing and generation of transgenes

All fly stocks were maintained at 22°C. The *ham^PRΔ^* mutants were created using CRISPR: transgenic flies carrying single-guide RNA targeting the PR domain containing isoforms were crossed into the vas-Cas9 transgenic flies. Frame-shift mutations were identified by PCR and Sanger sequencing. Transgenic flies were created at BestGene, Inc.

The ham Gal4 lines and RNAi stocks were obtained from Bloomington Stock Center. Knocking down experiments were performed at 25°C and with a regular 12-hr light–dark cycle.

To generate ham null mutant somatic clones in the TE terminal epithelium, *hamsk1 FRT40A/CTG* males were crossed with virgin females carrying the genotype *hsFLP(X); hsGFP.nls FRT40A*. Embryos aged 0–5 hr were collected and developed at 25°C until the second instar larval stage. GFP-negative larvae, indicating the genotype *hsFLP(X); hsGFP.nls/hamsk1 FRT40A*, were then selected. Head shock treatments at 37°C for 1 hr were administered three times during the second and third instar larval stages to induce somatic clones. Prior to dissection, another 1 hr head shock at 37°C was performed to induce GFP expression, marking the wild-type cells.

### Fertility test

For ham mutants, single male or female with indicated genotype was mated with two w^1118^ virgin females or two w^1118^ males on day 0 of the experiment. Crosses were flipped every 2 days for three consecutive times. Progenies from the second vial with eggs laid between days 2 and 4 were counted after 2 weeks.

For the RNAi screen, individual UAS-RNAi males were crossed with females carrying UAS-dcr2 and hamRSGal4. The resultant F1 male progenies (2–5 days old) with the genotype UAS-dcr2; hamRSGal4>UAS.RNAi were crossed to w^1118^ virgin females (1–2 days old) on day 0 of the experiment. In each test, four males were crossed with three females. Crosses were flipped on days 2 and 3 for pre-laying. After flipping on day 3, crosses were kept in the new vial for egg laying for a specific time period (Group A: 48 hr; Group B: 16–18 hr; Group C: 7–8 hr). If at least three males and two females were alive, then the flies were transferred to a new vial, the old vial was kept, and all progenies were counted.

For fertility test of the Wnt2 and Tl signaling pathways components, UAS-RNAi males were crossed to virgin females of the genotype UAS-dcr2; hamRSGal4. Two F1 male progenies (1–2 days old) with the genotype UAS-dcr2; hamRSGal4>UAS.RNAi were crossed to three w^1118^ virgin females (1–2 days old) on day 0 of the experiment. Crosses were flipped every day for four consecutive times. Progenies from the fourth vial with eggs laid between days 3 and 4 were counted after 2 weeks.

### Immunostaining

For embryo staining: embryos were collected, dechorionated with bleach, and fixed with 4% formaldehyde in PEM (0.1 M PIPES, 2 mM EGTA, 1 mM MgSO_4_, pH 7) for 20 min. For immunostaining, embryos were rehydrated, washed with PBT (0.3% Triton X-100 in PBS), and blocked with PBSBT (0.3% Triton X-100, 1% BSA in PBS).

For tissue staining: tissues were dissected from stage defined larvae or pupae in 1× PBS within 30 min and fixed in 4% paraformaldehyde in PBS for 20 min. For immunostaining, dissected tissues were washed with PBT (0.1% Triton X-100 in PBS) and blocked with PBSBT (0.1% Triton X-100, 1% BSA in PBS).

For both embryo and tissue staining, the primary antibodies were diluted with PBSBT (0.1% Triton X-100, 1% BSA in PBS) and incubated at 4°C over night. After extensive washing with PBSBT (15 min × 4 times), the secondary antibodies were added in 1:500 dilution and incubated at room temperature for 2 hr. After washing with PBT (0.1% Triton X-100 in PBS), the samples were counter-stained with DAPI and mounted in Vectashield (Vector Laboratories).

The following primary antibodies were used: Rabbit anti-Ham (1:300) (Gift from Eric Lai’s lab), Ms anti-V5 (1:500, R960-25, invitrogen), chk anti-GFP (1:2000, ab13970, Abcam), Rat anti-vasa (1:50, anti-vasa-c, Developmental Studies Hybridoma Bank, DSHB), Ms anti-ABD-B (1:50, 1A2E9-c), Ms anti-SXL (1:50, M18-c), rat anti-DE-Cad (1:50, DCAD2, DSHB), mouse anti-Crb (1:10, Cq4 DSHB), and mouse anti-Coracle (1:100, C615.16 DSHB). Secondary antibodies conjugated to Cy3 or Cy5 or Alexa Fluor-488 (Jackson ImmunoResearch) were used.

### Quantification of signal intensity from fluorescent immunostaining

For E-Cad and Crb in Figure 2, ‘raw intensity density’ from manually defined regions (using free-hand drawing) along the apical side of the SV and TE terminal epitheliums was obtained in ImageJ. For each sample, two regions in the SV and three regions in the TE terminal epithelium were averaged. The regions used to determine relative E-Cad fluorescence in *hamSK1* TE somatic clones were at the apical side between cell junction. The length of Cor-stained cell junction was measured using ImageJ. A straight line was drawn over the Cor staining with the ‘Freehand Line tool’. For each sample, the average length from 10 cell–cell junctions were used. In all experiments, images for the genotypes in comparison were always processed using exact settings. At least three biological replicates were included in statistical analysis.

### Western blot

Embryos from *w1118, ham^PRΔ^/CTG, ham^SK1^/CTG* were collected and aged to embryonic stage S13 to S16. In each blot, 50–100st GFP+ or GFP− embryos were used. The selected embryos were homogenized directly in 1xSDS loading buffer (62.5 mM Tris-HCl pH 6.8, 2.5% SDS, 0.002% Bromophenol Blue, 0.7135 M (5%) β-mercaptoethanol, 10% glycerol) and denatured at 98°C heat blot for 5 min. After centrifuged, supernatant was taken for western blotting with 8% of SDS–PAGE. Rabbit anti-Ham (1:2000) primary antibody was used in blotting. To minimize unspecific signal from IgG heavy chains, light chain-specific secondary antibody of mouse anti-Rabbit IgG (1:40,000 Jackson ImmunoResearch) was used, and the signals were developed with ECL Plus reagent (GE Healthcare).

### Cell culture and Luciferase assays

To generate different variants of Ham, the ORFs of HamFL, Ham-NZF-vp16, Ham-CZF-vp16, and *ham^PRΔ^* were PCR amplified and cloned into the pAC_V5 (nitrogen) vector. The enhancer fragments were cloned in to the p2xTetO-Firefly luciferase vector. All transfections were performed using *Drosophila* S2-R+ cells grown in Schneider *Drosophila* medium containing 10% fetal calf serum. Cells were co-transfected with the Ham expression construct, 2xTetO-Firefly luciferase and pAc-Renilla plasmids in 96-well plate using the Effectene Transfection kit (QIAGEN). Luciferase assays were performed and measured using the Dual Luciferase Assay System (Promega). Expression was calculated as the ratio between the firefly and *Renilla* luciferase activities. Three rounds of transfections were performed on different days and different cell populations and considered as three biological replicates. Within each biological replicate, four wells of cells were transfected with the same DNA mixture and measured and thus considered as four technique replicates. In total, for each DNA combination, the values from the 12 wells of transfection were calculated, averaged and plotted in Figure 4F, G and Figure 3—figure supplement 1D.

#### SCRINSHOT

The *Drosophila* RS was dissected at defined ages in 1× PBS and fixed in 4% paraformaldehyde in PBS for 20 min. After washing with PBST 0.1% (Triton X-100) for 2 × 15 min, fine dissected samples were placed on Poly-lysine coverslip and freezed at –80°C after drying on a heater.

The SCRINSHOT experiments were carried out according to the published method (Sountoulidis et al., 2020). In brief, three padlock probes and three corresponding detection probes were designed for each gene of interest. The slides with fine dissected samples were pretreated at 45°C to reduce moisture and fixed in 4% PFA in 1× PBS, followed by washing in PBS Tween-20 0.05% twice. Samples were then dehydrated with 70%, 85%, 100% ETOH and air dry. The SecureSeal hybridization chamber (GRACE BIO-LABS, 621501) was then mounted to cover samples on the slide. Permeabilization of tissues were first done by treating with 10 µg/ml Proteinase K (Invitrogen) in PBSTween-20 0.05% for 7 min followed by washing with glycine (2 mg/ml in PBS Tween-20 0.05%) once and with PBS Tween-20 0.05% for 3 × 5 min. After this, samples were further permeabilized in 0.1 M HCl for 3 min, followed by washing in PBS Tween-20 0.05% twice. Samples were then blocked in a probe-free hybridization reaction mixture of 1× Amplifase buffer (Lucigen, A1905B), 0.05 M KCl, 20% formamide deionized (Millipore S4117), 0.1 µM Oligo-dT, 0.1 µg/µl BSA (New England Biolabs, B9000S), 1 U/µl RiboLock (Thermo, EO0384), and 0.2 µg/µl tRNAs (Ambion, AM7119). Then hybridization of padlock probes was done by incubating samples with padlock probes (with the concentration of each one 0.01 µM) mixed in blocking reagents used before (no oligo dT used in this step). The slide was then put into a PCR machine to denature at 55°C for 15 min and hybridize at 45°C for 120 min. Padlock probes were then ligated by using SplintR ligase (NEB M0375) at 25°C for 16 hr followed by RCA (rolling cycle amplification) at 30°C for 16 hr by using phi29 polymerase (Lucigen, 30221-2) and RCA primer1. Then a fixation step was applied to stabilize RCA product in 4% PFA for 15 min followed by washing in PBS Tween-20 0.05%. Then the hybridization of the first three genes was done by mixing all 3,3′ fluorophore-conjugated detection probes of each gene in reaction reagent 2× SSC, 20% Formamide deionized, 0.1 µg/µl BSA, and 0.5 ng/μl DAPI (Biolegend, 422801) followed by hybridization at 30°C for 1 hr. The slides were washed in 20% formamide in 2× SSC and then in 6× SSC, followed by dehydration in 70%, 85%, and 100% ethanol until the chamber was removed. Then the samples were preserved in SlowFade Gold Antifade mountnant (Thermo, S36936) and kept in dark before imaging. After image acquisition, the first three detection probes were removed by using Uracil-DNA Glycosylase (Thermo, EN0362), and the slides were ready for the next round of detection probe hybridization. The procedure was repeated until all genes were hybridized and imaged. Images were acquired with an airyscan-equipped confocal microscope system (Zeiss LSM 800, Carl Zeiss), and ZEN 2.5 Blue edition software was used for image processing.

### Quantification of SCRINSHOT signals

Similar to quantification of immunofluorescent signal, fluorescence intensity of SCRINSHOT for each gene was measured in defined areas within images of the SV, EjD, and TE in 2D projections. Squares were drawn to define areas at the leading side of the SV, the middle region of the Ejd and the TE terminal. The total fluorescence intensity was first normalized to area size. The resultant value for *ham* mutant or RNAi samples was then contrasted to the normalized intensity of control samples.

#### CUT&TAG

CUT&TAG was performed according to the published method (Kaya-Okur et al., 2019). In brief, TE and grossly tissue of the posterior abdomen were dissected from 18 to 25 hr APF *w1118* and *pBAC_ham_sfGFP* animals in PBS within 30 min. For grossly tissues, the dissected samples were homogenized in 2 ml NE1 buffer followed by incubation on ice for 10 min. After filtering out the grossly cuticles, nuclei were collected by spinning at 1300 × *g* and resuspended in 1 ml PBS. For TE, nuclei extraction was done by simply pipetted samples in 200 µl NE1 up and down for 20–30 times and incubated on ice for 10 min. Light fixation was performed with 0.1% formaldehyde at RT for 2 min and neutralized with 75 mM glycine. Then the nuclei were washed by PBS and wash buffer one time each and kept in 200 µl wash buffer on ice until enough samples were collected (30–50st animals per/antibody). Resuspended nuclei in 1.2 ml wash buffer and mixed with 20 µl Concanavalin A-coated magnetic beads (Polyscience 86057) with soft vortexing. Aliquoted samples into two parts immediately and incubated for 10 min with rotation to allow the binding of the nuclei with the beads. The nuclei/beads mix was then blocked with 1 ml cold antibody buffer for 10 min and resuspended in 150 µl cold antibody buffer. For w1118 samples, 1 µl Rabbit anti-Ham or 1 µl Rabbit IgG (Sigma-Aldrich, I5006) was added for an overnight incubation at 4°C. For *pBAC_ham_sfGFP* samples, 1 µl Rabbit anti-GFP (G10362, Invitrogen) or Rabbit IgG was used. The beads/nuclei/antibody mix was then washed with Dig-wash buffer and incubated with secondary antibody (1:100 dilution) for 1 hr. After further washes with the Dig-wash buffer, a pA-Tn5 adaptor complex in Dig-300 buffer was added to the beads for 1 hr reaction. The beads were then washed with Dig-300 buffer before the incubation with 300 µl Tagmentation buffer at 37°C for 1 hr. Then 10 µl 0.5 M EDTA, 3 µl 10% SDS, and 2.5 µl 20 mg/ml proteinase K (Invitrogen) were used to stop the reaction. Then the resultant DNA was purified with DNA clean & Concentrator kit (ZYMO Research D4013) and eluted in 25 µl Elution buffer. To generate libraries, 21 µl fragment DNA was mixed with 2 µl 10 µM Universal i5 primer, 2 µl 10 µM uniquely barcoded i7 primer and 25 µl PCR master mix (NEB Phusion High-Fidelity PCR Master Mix with HF Buffer, M0531). The PCR condition was as follows: 72°C 5 min, 98°C 30 s, repeat 12 times (98°C 10 s, 63°C 10 s), 72°C 1 min and hold 4°C. The libraries were cleaned with standard Ampure XP beads as previously described. Libraries from four biological replicates were produced for each condition.

We mapped the CUT&TAG samples of *w1118* and *pBAC_ham_sfGFP* in TE and TG tissues to the dm6 genome assembly using Bowtie2 with the following parameters: bowtie2 --end-to-end --very-sensitive --no-mixed --no-discordant --phred33 -I 10 -X 700. The coverage for these samples was generated using bedtools genomeCoverageBed and normalized to library depth to yield reads per million per base. For peak calling, we employed SEACR (Meers et al., 2019), which is tailored for sparse chromatin profiling data like CUT&TAG. We first used a relaxed FDR cutoff of <0.5 for each replicate (SEACR_1.3.sh normalized_coverage 0.5 norm stringent output_peaks), we then identified common peaks across replicates for each condition. To select confident peaks for *w1118* and *pBAC_ham_sfGFP*, we overlapped their peaks within TE and TG separately, choosing the top 1800 peaks from TE and the top 6000 from TG. Peak annotation was performed by HOMER’s annotatePeaks.pl. Peak overlap analysis was performed using HOMER’s mergePeaks function with the default -d given parameter, ensuring that only directly overlapping peaks were merged. This method preserved the integrity of the original peak boundaries and maintained the specified distances between peaks from the initial peak calls. De novo motif discovery for peaks in up- or downregulated genes, positioned 2000 bp upstream and 500 bp downstream of the TSS in TE and TG tissues, was performed using the MEME-ChIP software. The parameters used were: -ccut 100, -meme-p 5, -dna, -meme-mod anr, -minw 5, -maxw 15, and -filter-thresh 0.05.

#### RNA-seq

Total RNA was extracted from finely dissected GDs or testis samples from 18 to 25 hr APF animals using Trizol reagent (Invitrogen). RNA quality was tested by Agilent Bioanalyzer.

RNA-seq libraries were made using the Illumina Truseq Total RNA library Prep Kit LT. Sequencing was performed on the Illumina Hiseq2500 platform. Following the trimming of adapter sequences with Trimmomatic, RNA-seq reads from replicated wild type (×3) and mutant samples (×3) were aligned to the *Drosophila melanogaster* (dm6) genome assembly utilizing HISAT2. Gene annotations were sourced from UCSC. The DESeq2 Bioconductor package was employed to identify differentially expressed mRNAs between *w1118* and the *ham^PRΔ^*/*ham^PRΔ^* mutant, as well as between +/Df and *ham^SK1^*/Df mutant, with a subsequent estimation of the FDR. Genes with an FDR <10% and absolute log_2_FC >0 were considered differentially expressed. Details of the calculated differential expression are provided in Supplementary file 4, table 4.

#### STRING gene network analyses

Ham activated (genes downregulated in *hamSK1* and bound by Ham in TG) and repressed (genes upregulated in *hamSK1* and bound by Ham in TG) gene networks are constructed using String, MCL clustering find natural clusters the stochastic flow, inflation parameter, 2. Disconnected genes are hidden. The position of the clusters is manually adjusted to fit related GO categories. The links to the gene networks in STRING: https://string-db.org/cgi/network?taskId=bbF8IgMTqf1M&sessionId=bzDIAZvMgWsi; https://string-db.org/cgi/network?taskId=bhKCcMoLlbwh&sessionId=bzDIAZvMgWsi

## Data Availability

All sequencing data produced in this study have been submitted to Gene Expression Omnibus (accession number: GSE290310). All newly produced materials from this study are available for the research community. The following dataset was generated: DaiQ WenJ WangH 2025*Drosophila* Hamlet mediates epithelial tissue assembly of the reproductive systemNCBI Gene Expression OmnibusGSE29031010.7554/eLife.104164PMC1222720240613556

## Additional files

Supplementary file 1. Clusters and genes from STRING network analysis.

Supplementary file 2. Genes that showed male sterility in the RNAi screen.

Supplementary file 3. Tested genes and their expression in SCRINSHOT.

Supplementary file 4. Differentially expressed genes from RNA-seq data.

MDAR checklist

## Appendix 1

**Appendix 1—key resources table:**

Reagent type (species) or resource	Designation	Source or reference	Identifiers	Additional information
Genetic reagent (*D. melanogaster*)	*w1118*	Bloomington *Drosophila* Stock Center	BDSC:3605 FLYBase: FBst0003605; RRID:BDSC_3605	FlyBase symbol: *w^1118^*
Genetic reagent (*D. melanogaster*)	*hamPR Δ FRT40A/Cyo*	This paper		A new hamlet mutant fly line
Genetic reagent (*D. melanogaster*)	*hamSK1 FRT40A/Cyo*	This paper		A new hamlet mutant fly line
Genetic reagent (*D. melanogaster*)	*ham1,FRT40A/Cyo*	Moore et al., 2002 DOI: 10.1126/science.1072387		
Genetic reagent (*D. melanogaster*)	*pBAC_ham_sfGFP*	Bloomington *Drosophila* Stock Center	BDSC:83660 FLYBase: FBst0083660; RRID:BDSC_83660	FlyBase symbol: *y1 w*; PBac {ham-GFP.FPTB}VK00033/TM6C, Sb1*
Genetic reagent (*D. melanogaster*)	*hamRSGal4*	Bloomington *Drosophila* Stock Center	BDSC:48361 FLYBase: FBst0048361; RRID:BDSC_48361	FlyBase symbol: *w1118;P {GMR80G06-GAL4}attP2*
Genetic reagent (*D. melanogaster*)	*hamRNAi*	Bloomington *Drosophila* Stock Center	BDSC:26728 FlyBase: FBst0026728; RRID:BDSC_26728	FlyBase symbol: *y1 v1;P{TRiP.JF02270}attP2*
Genetic reagent (*D. melanogaster*)	*UAS-GFP*	Bloomington *Drosophila* Stock Center	BDSC:4775 FlyBase: FBst0004775; RRID:BDSC_4775	FlyBase symbol: *w1118; P{UAS-GFP.nls}14*
Genetic reagent (*D. melanogaster*)	*GFP.RNAi*	Bloomington *Drosophila* Stock Center	BDSC:9330 FlyBase: FBst0009330; RRID:BDSC_9330	FlyBase symbol: *w1118;P{UAS-GFP.RNAi.R}142*
Genetic reagent (*D. melanogaster*)	*Wnt2O*	Bloomington *Drosophila* Stock Center	BDSC:6958 FlyBase: FBst0006958; RRID:BDSC_6958	FlyBase symbol: *Wnt2O/CyO, amosRoi-1*
Genetic reagent (*D. melanogaster*)	*UASWnt2*	Bloomington *Drosophila* Stock Center	BDSC:6961 FlyBase: FBst0006961; RRID:BDSC_6961	FlyBase symbol:
Genetic reagent (*D. melanogaster*)	*fz2.RNAi*	Bloomington *Drosophila* Stock Center	BDSC:27568 FlyBase: FBst0027568; RRID:BDSC_27568	FlyBase symbol: *y1 v1;P{TRiP.JF02722}attP2*
Genetic reagent (*D. melanogaster*)	*fz2.RNAi*	Bloomington *Drosophila* Stock Center	BDSC: 67863 FlyBase: FBst0067863; RRID:BDSC_67863	FlyBase symbol: *y1 sc* v1 sev21; P{TRiP.HMS05675}attP40*
Genetic reagent (*D. melanogaster*)	*fz.RNAi*	Vienna *Drosophila* Resource Center	VDRC: 43075 FlyBase: FBst0464905;	FlyBase symbol: *w1118;P{GD4614}v43075*
Genetic reagent (*D. melanogaster*)	*Toll-9.RNAi*	Bloomington *Drosophila* Stock Center	BDSC: 34853 FlyBase: FBst0034853; RRID:BDSC_34853	FlyBase symbol*: y1 sc* v1 sev21; P{TRiP.HMS00171}attP2*
Genetic reagent (*D. melanogaster*)	*pbl.RNAi*	Bloomington *Drosophila* Stock Center	BDSC: 28343 FlyBase: FBst0028343; RRID:BDSC_28343	FlyBase symbol: *y1 v1;P{TRiP.JF02979}attP2*
Genetic reagent (*D. melanogaster*)	*CG11406.RNAi*	Bloomington *Drosophila* Stock Center	BDSC: 61185 FlyBase: FBst0061185; RRID:BDSC_61185	FlyBase symbol: *y1 v1;P{TRiP.HMJ22905}attP40*
Genetic reagent (*D. melanogaster*)	*AP-2alpha.RNAi*	Bloomington *Drosophila* Stock Center	BDSC: 32866 FlyBase: FBst0032866; RRID:BDSC_32866	FlyBase symbol: *y1 sc* v1 sev21; P{TRiP.HMS00653}attP2*
Genetic reagent (*D. melanogaster*)	*shg.RNAi*	Bloomington *Drosophila* Stock Center	BDSC: 32904 FlyBase: FBst0032904; RRID:BDSC_32904	FlyBase symbol: *y1 sc* v1 sev21; P{TRiP.HMS00693}attP2*
Genetic reagent (*D. melanogaster*)	*GstE11.RNAi*	Bloomington *Drosophila* Stock Center	BDSC: 82975 FlyBase: FBst0082975; RRID:BDSC_82975	FlyBase symbol: *y1 sc* v1 sev21; P{TRiP.HMC06653}attP40*
Genetic reagent (*D. melanogaster*)	*CG7456.RNAi*	Bloomington *Drosophila* Stock Center	BDSC: 51866 FlyBase: FBst0051866; RRID:BDSC_51866	FlyBase symbol: *y1 v1;P{TRiP.HMC03440}attP40*
Genetic reagent (*D. melanogaster*)	*CG7456.RNAi*	Bloomington *Drosophila* Stock Center	BDSC: 53679 FlyBase: FBst0053679; RRID:BDSC_53679	FlyBase symbol: *y1 v1;P{TRiP.HMJ21592}attP40*
Genetic reagent (*D. melanogaster*)	*Gr64c.RNAi*	Bloomington *Drosophila* Stock Center	BDSC: 36734 FlyBase: FBst0036734; RRID:BDSC_36734	FlyBase symbol: *y1 sc* v1 sev21;P{TRiP.HMS01625}attP40*
Genetic reagent (*D. melanogaster*)	*AkhR.RNAi*	Bloomington *Drosophila* Stock Center	BDSC: 29577 FlyBase: FBst0029577; RRID:BDSC_29577	FlyBase symbol: y1v1;P{TRiP.JF03256}attP2
Genetic reagent (*D. melanogaster*)	*AkhR.RNAi*	Bloomington *Drosophila* Stock Center	BDSC: 51710 FlyBase: FBst0051710; RRID:BDSC_51710	FlyBase symbol: y1v1*; P{TRiP.HMC03228}attP40*
Genetic reagent (*D. melanogaster*)	*Idgf4.RNAi*	Bloomington *Drosophila* Stock Center	BDSC: 55381 FlyBase: FBst0055381; RRID:BDSC_55381	FlyBase symbol: *y1 sc* v1 sev21; P{TRiP.HMC04069}attP40*
Genetic reagent (*D. melanogaster*)	*CG17477.RNAi*	Bloomington *Drosophila* Stock Center	BDSC: 42821 FlyBase: FBst0042821; RRID:BDSC_42821	FlyBase symbol: *y1 v1;P{TRiP.HMS02503}attP40*
Genetic reagent (*D. melanogaster*)	*CG11069.RNAi*	Bloomington *Drosophila* Stock Center	BDSC: 77158 FlyBase: FBst0077158; RRID:BDSC_77158	FlyBase symbol: *y1 sc* v1 sev21; P{TRiP.HMS05930}attP40*
Genetic reagent (*D. melanogaster*)	*CG17186.RNAi*	Bloomington *Drosophila* Stock Center	BDSC: 27079 FlyBase: FBst0027079; RRID:BDSC_27079	FlyBase symbol: *y1 v1;P{TRiP.JF02425}attP2*
Genetic reagent (*D. melanogaster*)	*CG4872.RNAi*	Bloomington *Drosophila* Stock Center	BDSC: 64486 FlyBase: FBst0064486; RRID:BDSC_64486	FlyBase symbol: *y1 v1;P{TRiP.HMC05503}attP40*
Genetic reagent (*D. melanogaster*)	*AdSL.RNAi*	Bloomington *Drosophila* Stock Center	BDSC: 34347 FlyBase: FBst0034347; RRID:BDSC_34347	FlyBase symbol: *y1 sc* v1 sev21; P{TRiP.HMS01336}attP2*
Genetic reagent (*D. melanogaster*)	*Tl.RNAi*	Bloomington *Drosophila* Stock Center	BDSC: 31044 FlyBase: FBst0031044; RRID:BDSC_31044	FlyBase symbol: *y1 v1;P{TRiP.JF01491}attP2*
Genetic reagent (*D. melanogaster*)	*CG17283.RNAi*	Bloomington *Drosophila* Stock Center	BDSC: 63723 FlyBase: FBst0063723; RRID:BDSC_63723	FlyBase symbol: *y1 v1;P{TRiP.HMJ30291}attP40/CyO*
Genetic reagent (*D. melanogaster*)	*decay.RNAi*	Bloomington *Drosophila* Stock Center	BDSC:65879 FlyBase: FBst0065879; RRID:BDSC_65879	FlyBase symbol: *y1 sc* v1 sev21; P{TRiP.HMC06141}attP2*
Genetic reagent (*D. melanogaster*)	*PH4alphaMP.RNAi*	Bloomington *Drosophila* Stock Center	BDSC: 65190 FlyBase: FBst0065190; RRID:BDSC_65190	FlyBase symbol: *y1 sc* v1 sev21; P{TRiP.HMC06065}attP40*
Genetic reagent (*D. melanogaster*)	*Hr46.RNAi*	Bloomington *Drosophila* Stock Center	BDSC: 27253 FlyBase: FBst0027253; RRID:BDSC_27253	FlyBase symbol: *y1 v1;P{TRiP.JF02542}attP2*
Genetic reagent (*D. melanogaster*)	*CG15117.RNAi*	Bloomington *Drosophila* Stock Center	BDSC: 33693 FlyBase: FBst0033693; RRID:BDSC_33693	FlyBase symbol: *y1 sc* v1 sev21; P{TRiP.HMS00562}attP2*
Genetic reagent (*D. melanogaster*)	*gsb-n.RNAi*	Bloomington *Drosophila* Stock Center	BDSC: 28078 FlyBase: FBst0028078; RRID:BDSC_28078	FlyBase symbol: *y1 v1;P{TRiP.JF02915}attP2*
Genetic reagent (*D. melanogaster*)	*shark.RNAi*	Bloomington *Drosophila* Stock Center	BDSC: 25788 FlyBase: FBst0025788; RRID:BDSC_25788	FlyBase symbol: *y1 v1;P{TRiP.JF01794}attP2*
Genetic reagent (*D. melanogaster*)	*CG17018.RNAi*	Bloomington *Drosophila* Stock Center	BDSC: 57024 FlyBase: FBst0057024; RRID:BDSC_57024	FlyBase symbol: *y1 sc* v1 sev21; P{TRiP.HMS04468}attP40*
Genetic reagent (*D. melanogaster*)	*cag.RNAi*	Bloomington *Drosophila* Stock Center	BDSC: 63554 FlyBase: FBst0063554; RRID:BDSC_63554	FlyBase symbol: *y1 v1;P{TRiP.HMJ30120}attP40/CyO*
Genetic reagent (*D. melanogaster*)	*CG7484.RNAi*	Bloomington *Drosophila* Stock Center	BDSC: 61869 FlyBase: FBst0061869; RRID:BDSC_61869	FlyBase symbol: *y1 v1;P{TRiP.HMJ23358}attP40*
Genetic reagent (*D. melanogaster*)	*CG31935.RNAi*	Bloomington *Drosophila* Stock Center	BDSC: 64495 FlyBase: FBst0064495; RRID:BDSC_64495	FlyBase symbol: *y1 sc* v1 sev21; P{TRiP.HMC05513}attP40*
Antibody	Rabbit anti-Ham	Gift from Eric Lai’s lab		IF (1:300), WB (1:2000) Cut&Tag (1:150)
Antibody	Rabbit anti-GFP	Invitrogen	Cat# G10362	Cut&Tag (1:150)
Antibody	IgG from rabbit serum	Sigma-Aldrich	Cat# I5006-10MG	Cut&Tag (1:150)
Antibody	guineapig anti-rabbit	Antibodies online	Cat# ABIN101961	Secondary antibody for Cut&Tag (1:100)
Antibody	Mouse anti-V5	Invitrogen	Cat# R960-25	IF (1:500)
Antibody	chicken anti-GFP	Abcam	Cat# ab13970	IF (1:2000)
Antibody	Rat anti-vasa	Developmental Studies Hybridoma Bank, DSHB	Cat# anti-vasa-c	IF (1:50)
Antibody	Mouse anti-ABD-B	DSHB	Cat# 1A2E9-c	IF (1:50)
Antibody	Mouse anti-SXL	DSHB	Cat# M18-c	IF (1:50)
Antibody	Rat anti-E-Cad	DSHB	Cat# DCAD2	IF (1:50)
Antibody	Mouse anti-Crb	DSHB	Cat# Cq4	IF (1:10)
Antibody	Mouse anti-Cor	DSHB	Cat# C615.16	IF (1:100)
antibody	Secondary antibodies conjugated to Cy3 or Cy5 or Alexa Fluor-488	Jackson ImmunoResearch		IF (1:1000)
Antibody	mouse anti-Rabbit IgG light chain specific	Jackson ImmunoResearch	Cat# 211-032-171	WB (1:40,000)
Sequence-based reagent	hamPRΔ_CRISPR gRNA	This paper	gRNA sequence for CRISPR/Cas9	TCAGGACGATTGTATAGGCG
Sequence-based reagent	hamPRΔ check_Forward	This paper	PCR primers	AACACTTTTG AGGGTTGTGTCTTG
Sequence-based reagent	hamPRΔ check_Reverse	This paper	PCR primers	ACAGGACTCTTGGGTCGCCC
Sequence-based reagent	DWnt2_Luc1_F	This paper	PCR primers	TTGGCGCGCCACTCCTCCCTCCATTTGGCCAC
Sequence-based reagent	DWnt2_Luc1_R	This paper	PCR primers	TTGGCGCGCCACCTTCTTCTGCTCCAGCCAACG
Sequence-based reagent	DWnt2_Luc2_F	This paper	PCR primers	TTGGCGCGCCGCTTGAAAAGGATCCGTGATC
Sequence-based reagent	DWnt2_Luc2_R	This paper	PCR primers	TTGGCGCGCCCCATCATTGATGGAAGGTC
Sequence-based reagent	DWnt2_Luc3_F	This paper	PCR primers	TTGGCGCGCCCTCGCAGTGTCGTACATATGTC
Sequence-based reagent	DWnt2_Luc3_R	This paper	PCR primers	TTGGCGCGCCTGAGATCACATGCAATGACGC
Sequence-based reagent	DWnt2_Luc4_F	This paper	PCR primers	TTGGCGCGCCGCGTCATTGCATGTGATCTC
Sequence-based reagent	DWnt2_Luc4_R	This paper	PCR primers	TTGGCGCGCCGCGGCAATAATCATGCGATC
Sequence-based reagent	shot_Luc1_F	This paper	PCR primers	TTGGCGCGCCAGTGCATGGATGCTAGGCGAAC
Sequence-based reagent	shot_Luc1_R	This paper	PCR primers	TTGGCGCGCCAACCAGCTTGTGCCTGCTACG
Sequence-based reagent	shot_Luc2_F	This paper	PCR primers	TTGGCGCGCCAGGTGTATAGAGCGGTGCATG
Sequence-based reagent	shot_Luc2_R	This paper	PCR primers	TTGGCGCGCCCAGTGCGCTTTATATCGCTCG
Sequence-based reagent	shg_Luc1_F	This paper	PCR primers	TTGGCGCGCCCAACTCGAACTCGACTCAGTGG
Sequence-based reagent	shg_Luc1_R	This paper	PCR primers	TTGGCGCGCCGTGTCGCTGGAACTCTTCCTTG
Sequence-based reagent	shg_Luc2_F	This paper	PCR primers	TTGGCGCGCCCTGCCACGAATGTTGACGATCC
Sequence-based reagent	shg_Luc2_R	This paper	PCR primers	TTGGCGCGCCCGATCTGACGACGACAGATGTC
Sequence-based reagent	ham_PL1	This paper	SCRINSHOT padlock probe	/5Phos/ATCCTGTATATCAAGATGATTCCTCTATGATTACTGACTGCGTCTATTTAGTGGAGCCGCCCCTATCTTCTTTGGTGAAGTAGCTCCTG
Sequence-based reagent	ham_PL2	This paper	SCRINSHOT padlock probe	/5Phos/ATGGTAGTTTCTGATCAAAGTCCTCT ATGATTACTGACTGCGTCTATTTAGTGGAGCCGCCCCTATCTTCTTTTCACCCTTTAGAAAGCCAA
Sequence-based reagent	ham_PL3	This paper	SCRINSHOT padlock probe	/5Phos/CGAAGTACTCCTCCCTTTCCTCTATGATTACTGACTGCGTCTATTTAGTGGAGCCGCCCCTATCTTCTTTCATGAACTTCCTTATGTCAT
Sequence-based reagent	wnt2_PL1	This paper	SCRINSHOT padlock probe	/5Phos/AGCTCATTAGTGACGCAATTTCCTCTATGATTACTGACTGCGTCTATTTAGTGGAGCCGCCCCTATCTTCTTTGCGGAGATTAACATAAATGC
Sequence-based reagent	wnt2_PL2	This paper	SCRINSHOT padlock probe	/5Phos/TATCGCCAACCAGTCGAATCCTCTATGATTACTGACTGCGTCTATTTAGTGGAGCCGCCCCTATCTTCTTTTTGATACTTCAGCATGAGTC
Sequence-based reagent	wnt2_PL3	This paper	SCRINSHOT padlock probe	/5Phos/GACATAGCCCTAGTTCTAGTCCTCTATGATTACTGACTGCGTCTATTTAGTGGAGCCGCCCCTATCTTCTTTCTTAATCAACGCATTTCCTAT
Sequence-based reagent	shg_PL1	This paper	SCRINSHOT padlock probe	/5Phos/ATCGAATCGAACTCGTACATCCTCTATGATTACTGACTGCGTCTATTTAGTGGAGCCGCCCCTATCTTCTTTCAACCGCTCAATAATTATCA
Sequence-based reagent	shg_PL2	This paper	SCRINSHOT padlock probe	/5Phos/CGATTATCGCATACGATTGGTCCTCTATGATTACTGACTGCGTCTATTTAGTGGAGCCGCCCCTATCTTCTTTACTACGTTATATGCCCGCA
Sequence-based reagent	shg_PL3	This paper	SCRINSHOT padlock probe	/5Phos/TTACGTTGATGAATCGGATGTCCTCTATGATTACTGACTGCGTCTATTTAGTGGAGCCGCCCCTATCTTCTTTCTGATAAACTCCTCCTTGG
Sequence-based reagent	shot_PL1	This paper	SCRINSHOT padlock probe	/5Phos/TCGCGGTAATCCTTGCATCCTCTATGATTACTGACTGCGTCTATTTAGTGGAGCCGCCCCTATCTTCTTTTATCAATAGTTGGTGCATAG
Sequence-based reagent	shot_PL2	This paper	SCRINSHOT padlock probe	/5Phos/TCCTTTAGGGTCTTCATTACTCCTCTATGATTACTGACTGCGTCTATTTAGTGGAGCCGCCCCTATCTTCTTTCAAGATAGTGTTTCCTCTAGA
Sequence-based reagent	shot_PL3	This paper	SCRINSHOT padlock probe	/5Phos/TATGCCATCGATGAAGACATTCCTCTATGATTACTGACTGCGTCTATTTAGTGGAGCCGCCCCTATCTTCTTTATGTATCGAATTTCGTATTGAG
Sequence-based reagent	pbl_PL1	This paper	SCRINSHOT padlock probe	/5Phos/TCGTAAACGTATAGAGCTTCTCATGATTACTGACTGCGTCTATTTAGAATATAAATGAGGTTGATGATGAGGACTTATCTGTCTCCTCATCACTGA
Sequence-based reagent	pbl_PL2	This paper	SCRINSHOT padlock probe	/5Phos/ACCGGATCGTATTCAAAGATATATGATTACTGACTGCGTCTATTTAGAATATAAATGAGGTTGATGATGAGGACTCTATCCGAGATGTCAATCACTA
Sequence-based reagent	pbl_PL3	This paper	SCRINSHOT padlock probe	/5Phos/CGAATAAACTATTTGAGACAGACAATGATTACTGACTGCGTCTATTTAGAATATAAATGAGGTTGATGATGAGGACTCGGACTGGTGGTACAAT
Sequence-based reagent	AP-2alpha_PL1	This paper	SCRINSHOT padlock probe	/5Phos/GCACTTGAGTTGTTGTTACTATCTATGATTACTGACTGCGTCTATTTACTCCTACACTCTTCTTAAGTACCTTTCCTTCTTCGTGTTGTACACG
Sequence-based reagent	AP-2alpha_PL2	This paper	SCRINSHOT padlock probe	/5Phos/TATGAATAACAGCTTACAGACATCTATGATTACTGACTGCGTCTATTTACTCCTACACTCTTCTTAAGTACCTTTCCATGGCCGAGGAGAAA
Sequence-based reagent	AP-2alpha_PL3	This paper	SCRINSHOT padlock probe	/5Phos/GCGACTGCAAATCATTCTTTACTAT GATTACTGACTGCGTCTATTTACTCCTACA CTCTTCTTAAGTACCTTTCGATTAACGTG CACAGGATTAC
Sequence-based reagent	CG11406_PL1	This paper	SCRINSHOT padlock probe	/5Phos/AATTTGCCGCTATTGATGAGTGAT CCTCTATGATTACTGACTGCGTCTATTTAG ATAGTGGTCCACTGTCCTGGGTAGTCGTACTGCTGG
Sequence-based reagent	CG11406_PL2	This paper	SCRINSHOT padlock probe	/5Phos/CCCGGCACCTGATACTGATCCTC TATGATTACTGACTGCGTCTATTTAGATAGTG GTCCACTGTCCTGGAACAGAAAGTCAATGTGATTATAG
Sequence-based reagent	CG11406_PL3	This paper	SCRINSHOT padlock probe	/5Phos/CTTATTCCTGTTTCCTCGTTCTGAT CCTCTATGATTACTGACTGCGTCTATTTAGA TAGTGGTCCACTGTCCTGGTCCACGTTTGGTGGAA
Sequence-based reagent	AkhR_PL1	This paper	SCRINSHOT padlock probe	/5Phos/CAAGGATTTGCCTCAAAGATAATCCTCT ATGATTACTGACTGCGTCTATTTACTGAGGAGA ATGATCATCGTGATGTATCTGAATGCAACTGCAT
Sequence-based reagent	AkhR_PL2	This paper	SCRINSHOT padlock probe	/5Phos/TCGCTTAATTGATCCAGTTGTCCTCTATG ATTACTGACTGCGTCTATTTACTGAGGAGAATGAT CATCGTGATTCCAGTTGAACATTAGCAGA
Sequence-based reagent	AkhR_PL3	This paper	SCRINSHOT padlock probe	/5Phos/AGATGCATTAGCATGATATCAATACTCCT CTATGATTACTGACTGCGTCTATTTACTGAGGAG AATGATCATCGTGATGATCGGCGATGGCC
Sequence-based reagent	CG17186_PL1	This paper	SCRINSHOT padlock probe	/5Phos/CAACATCTGGCTCTTCCTCTATGATTACT GACTGCGTCTATTTAGATAGAGGCTCTAGATTT CATCTACGTATCTATATCCTGTATATCCTCCA
Sequence-based reagent	CG17186_PL2	This paper	SCRINSHOT padlock probe	/5Phos/GATCTATTTGAACCACCTCGTCTATGAT TACTGACTGCGTCTATTTAGATAGAGGCTCTAG ATTTCATCTACGTATATTCTATCATTTCAAAGCCGG
Sequence-based reagent	CG17186_PL3	This paper	SCRINSHOT padlock probe	/5Phos/CAAGAGCTTCAGTTTCCTCTCTATGAT TACTGACTGCGTCTATTTAGATAGAGGCTCTAG ATTTCATCTACGTTCCAAGTATTCTACTGGCT
Sequence-based reagent	CG4872_PL1	This paper	SCRINSHOT padlock probe	/5Phos/CTGCTCATATTGGCAATGAAATCTCTAT GATTACTGACTGCGTCTATTTAGAAACTATCCC AAGGAGCTAGAAATGTAGTAAGCTGGCGTAGAG
Sequence-based reagent	CG4872_PL2	This paper	SCRINSHOT padlock probe	/5Phos/CGTGCGATTGTAAAGATTAAGCTCTATG ATTACTGACTGCGTCTATTTAGAAACTATCCCAA GGAGCTAGAAATCAGTGCCTTTGCCTG
Sequence-based reagent	CG4872_PL3	This paper	SCRINSHOT padlock probe	/5Phos/CTCTCGGTGTTCAATTTCTGCTCTATGA TTACTGACTGCGTCTATTTAGAAACTATCCCAAG GAGCTAGAAATATACAGTAATCTGGGTTCCG
Sequence-based reagent	decay_PL1	This paper	SCRINSHOT padlock probe	/5Phos/AAGAATTTGTCGAATGTGGAGATCCTCT ATGATTACTGACTGCGTCTATTTACTCAACCAGA TGAAGGAAATGCGTCAACGTTACGGAATGAG
Sequence-based reagent	decay_PL2	This paper	SCRINSHOT padlock probe	/5Phos/GTTTGTTCTTGAGCGTCTTATCCTCTATG ATTACTGACTGCGTCTATTTACTCAACCAGATGAA GGAAATGCCTGGATGAAGAAGAGTTTGG
Sequence-based reagent	decay_PL3	This paper	SCRINSHOT padlock probe	/5Phos/TCGTTGATCTCGGAGAAGATCCTCTATG ATTACTGACTGCGTCTATTTACTCAACCAGATGA AGGAAATGCCAACCTCTTTGAGCGTG
Sequence-based reagent	PH4alphaMP_PL1	This paper	SCRINSHOT padlock probe	/5Phos/CCTCCATAAAGTCTAGGGATATCCTCTAT GATTACTGACTGCGTCTATTTACAATGTATGGATT CGTGAACGCCGATGATATTCCACCACTG
Sequence-based reagent	PH4alphaMP_PL2	This paper	SCRINSHOT padlock probe	/5Phos/AAGTCGCGTAAGAGCTATCATCCTCTATG ATTACTGACTGCGTCTATTTACAATGTATGGATTC GTGAACGCCATCCAATTGATAAGTCTGCTG
Sequence-based reagent	PH4alphaMP_PL3	This paper	SCRINSHOT padlock probe	/5Phos/CTAAGATTTCTTTGCTTGGATGATCCTCTATGATTACTGACTGCGTCTATTTACAATGTATGGATTCGTGAACGCCTCTTCCTTAAGCGACAC
Sequence-based reagent	GstE11_PL1	This paper	SCRINSHOT padlock probe	/5Phos/AGACAGTGCTCCAAGCCTCTATGATCCTCTATGATTACTGACTGCGTCTATTTACTGGTGGATGCCCGTCAAGTAATCACCTTCAGCC
Sequence-based reagent	GstE11_PL2	This paper	SCRINSHOT padlock probe	/5Phos/CGACAATGAAACGGATTCGCTCTATGATCCTCTATGATTACTGACTGCGTCTATTTACTGGTGGATGCCCGTCGAAATAGATCACCGGCT
Sequence-based reagent	GstE11_PL3	This paper	SCRINSHOT padlock probe	/5Phos/ATATCCAGACCTTCCTGATTGCTCTATGATCCTCTATGATTACTGACTGCGTCTATTTACTGGTGGATGCCCGTTTAACCAGTCCCACCAAC
Sequence-based reagent	Cog4_PL1	This paper	SCRINSHOT padlock probe	/5Phos/GGATTCCAGTACTCCGATAGCCTCTATGATTACTGACTGCGTCTATTTACAATCAGTTCCTGAACTACAGTCAGATGACATCTCTTTGTTATTCTCC
Sequence-based reagent	Cog4_PL2	This paper	SCRINSHOT padlock probe	/5Phos/TGCACTATGAAGAACTGGACCTCTATGATTACTGACTGCGTCTATTTACAATCAGTTCCTGAACTACAGTCAGAGTTAAGTAGTCCATCCAAT
Sequence-based reagent	Cog4_PL3	This paper	SCRINSHOT padlock probe	/5Phos/CTTCTCATAGCGTTCCATTATATCCCTCTATGATTACTGACTGCGTCTATTTACAATCAGTTCCTGAACTACAGTCACAGATCGGAGTTCTTCATGAT
Sequence-based reagent	Gr64c_PL1	This paper	SCRINSHOT padlock probe	/5Phos/AAACTAAGAGATCCTATGGTATAGTGATCCTCTATGATTACTGACTGCGTCTATTTAGGCAAGGGATACTTTCTGCTGATGCAGATCACGCTG
Sequence-based reagent	Gr64c_PL2	This paper	SCRINSHOT padlock probe	/5Phos/GTTCTTGAATTGGAGATACGAGTGATCCTCTATGATTACTGACTGCGTCTATTTAGGCAAGGGATACTTTCTGCTCGTGACACTTATGACTAAATGC
Sequence-based reagent	Gr64c_PL3	This paper	SCRINSHOT padlock probe	/5Phos/GAAAGCTATGATAAATCTGCACTGATCCTCTATGATTACTGACTGCGTCTATTTAGGCAAGGGATACTTTCTGCTCATAGTTGCCCTTGGATT
Sequence-based reagent	Idgf4_PL1	This paper	SCRINSHOT padlock probe	/5Phos/CGTAGTCCAAGTTATTGATAATAGATGATCCTCTATGATTACTGACTGCGTCTATTTAGACTCAACGCAAACCTGTGGTAGGTGTGCAGGTTCA
Sequence-based reagent	Idgf4_PL2	This paper	SCRINSHOT padlock probe	/5Phos/AACAAAGTAGATGATGAGATCCATGATCCTCTATGATTACTGACTGCGTCTATTTAGACTCAACGCAAACCTGTGAACTGTTGCCGTCATAGT
Sequence-based reagent	Idgf4_PL3	This paper	SCRINSHOT padlock probe	/5Phos/GCTCTACAACTTGCTCTTAACATGATCCTCTATGATTACTGACTGCGTCTATTTAGACTCAACGCAAACCTGTGAAAGAATCAGAAGGATGAGGA
Sequence-based reagent	CG17477_PL1	This paper	SCRINSHOT padlock probe	/5Phos/CCTGAGTTAGTGCGTTAAAGATCCTCTATGATTACTGACTGCGTCTATTTAGATAATTACGGCTGGTCACTGTATGTGGGTAGTTCAACCG
Sequence-based reagent	CG17477_PL2	This paper	SCRINSHOT padlock probe	/5Phos/GGCAGACATCATGGACTATCCTCTATGATTACTGACTGCGTCTATTTAGATAATTACGGCTGGTCACTGTCAGTTCCAGATCCTCGTA
Sequence-based reagent	CG17477_PL3	This paper	SCRINSHOT padlock probe	/5Phos/TAAGCACAAATATGGCAAGGATCCTCTATGATTACTGACTGCGTCTATTTAGATAATTACGGCTGGTCACTGTTCCAATATTTGCCTGACGA
Sequence-based reagent	CG11069_PL1	This paper	SCRINSHOT padlock probe	/5Phos/AAATCGGAAATAAAGATATGGTAGCCTCTATGATTACTGACTGCGTCTATTTACAACTCTACTGATTGCCTACAACTGAGAACTTCTTGCGCC
Sequence-based reagent	CG11069_PL2	This paper	SCRINSHOT padlock probe	/5Phos/CTCTTATGCGCCACTTGCCTCTATGATTACTGACTGCGTCTATTTACAACTCTACTGATTGCCTACAACTCACTGATATTAAGATACTCCACT
Sequence-based reagent	CG11069_PL3	This paper	SCRINSHOT padlock probe	/5Phos/TGACCAACTGTATTCCAATAGCCTCTATGATTACTGACTGCGTCTATTTACAACTCTACTGATTGCCTACAACTCAATAGCATGACAGGATCTC
Sequence-based reagent	AdSL_PL1	This paper	SCRINSHOT padlock probe	/5Phos/CGTCTTGAACGTTCTTCAGTGATCCTCTATGATTACTGACTGCGTCTATTTACTCGAGTCGACGTTCTTGATCTAGATCTAGATGTTCAGTTTGA
Sequence-based reagent	AdSL_PL2	This paper	SCRINSHOT padlock probe	/5Phos/AGCCAGTAGCAACTGTTTTGATCCTCTATGATTACTGACTGCGTCTATTTACTCGAGTCGACGTTCTTGATCATAAGATATTCGGCTGCTTAA
Sequence-based reagent	AdSL_PL3	This paper	SCRINSHOT padlock probe	/5Phos/CGAGACATTGCTGGTTAGTGATCCTCTATGATTACTGACTGCGTCTATTTACTCGAGTCGACGTTCTTGATGTGATAATCCATTTAGCAACAG
Sequence-based reagent	Tl_PL1	This paper	SCRINSHOT padlock probe	/5Phos/GATCTGCACGTAGTCTTTGCTCTATGATTACTGACTGCGTCTATTTACTACAACAATAATCTGGTGCATGTGTATCCGTCAGATTGCACAT
Sequence-based reagent	Tl_PL2	This paper	SCRINSHOT padlock probe	/5Phos/TTAGTTCGAGATGACTCAGATTCCTCTATGATTACTGACTGCGTCTATTTACTACAACAATAATCTGGTGCATGTGCATTTCCTCGATATTTGCCC
Sequence-based reagent	Tl_PL3	This paper	SCRINSHOT padlock probe	/5Phos/TCCGAGTATATGATCACAATAATGCTCTATGATTACTGACTGCGTCTATTTACTACAACAATAATCTGGTGCATGTGCTTCTCCACATCTCCGATG
Sequence-based reagent	CG17283_PL1	This paper	SCRINSHOT padlock probe	/5Phos/TTGGTCCTCACCTTTAATTTCATCCTCTATGATTACTGACTGCGTCTATTTACAAGACCGAGTCAATCGTTAGAGACTGTCCTGCCTCTG
Sequence-based reagent	CG17283_PL2	This paper	SCRINSHOT padlock probe	/5Phos/ATACGTAGTGGCAGCGATCCTCTATGATTACTGACTGCGTCTATTTACAAGACCGAGTCAATCGTTAGAGTTAATACAGATAAGTCTAAGTGGA
Sequence-based reagent	CG17283_PL3	This paper	SCRINSHOT padlock probe	/5Phos/GGTCACGAAAGTGGTTCATCCTCTATGATTACTGACTGCGTCTATTTACAAGACCGAGTCAATCGTTAGACCTAGTATACCATCGAAATTTGA
Sequence-based reagent	Hr46_PL1	This paper	SCRINSHOT padlock probe	/5Phos/GGTATATTGTTCAGCAGTGTATGATTACTGACTGCGTCTATTTAGAACATAATCGAATTTGCTAAGCTCATACGGAAATATCGCGGAAATTG
Sequence-based reagent	Hr46_PL2	This paper	SCRINSHOT padlock probe	/5Phos/TCACTTTAACTGCTACTCAGATGATTACTGACTGCGTCTATTTAGAACATAATCGAATTTGCTAAGCTCATACGTATTCATTCGATTTCTCGC
Sequence-based reagent	Hr46_PL3	This paper	SCRINSHOT padlock probe	/5Phos/GGCAAAGACAACCTAGGATGATTACTGACTGCGTCTATTTAGAACATAATCGAATTTGCTAAGCTCATACCACATACTTTAGTTACAACGTAA
Sequence-based reagent	CG15117_PL1	This paper	SCRINSHOT padlock probe	/5Phos/GTGGTTATGTCATTATAGGATGCTATGATTACTGACTGCGTCTATTTACAAACCCATTATCATGTCTGAATATGGTGATCCCTCAAATTGTCC
Sequence-based reagent	CG15117_PL2	This paper	SCRINSHOT padlock probe	/5Phos/GATGTAGGTGAAGAGATCTTCCTATGATTACTGACTGCGTCTATTTACAAACCCATTATCATGTCTGAATATGGCATTAGGATATCAGATCGGC
Sequence-based reagent	CG15117_PL3	This paper	SCRINSHOT padlock probe	/5Phos/AGCTATGGAGAAGTGCAGCTATGATTACTGACTGCGTCTATTTACAAACCCATTATCATGTCTGAATATGGCTTTGTTGACCAGGATCAG
Sequence-based reagent	gsb-n_PL1	This paper	SCRINSHOT padlock probe	/5Phos/CCTCCAAGAATTCCATTTATGATGATTACTGACTGCGTCTATTTACAAATATAATTGTGCAATGGTATCGACTCCACTTATATCTGAGTCTCGC
Sequence-based reagent	gsb-n_PL2	This paper	SCRINSHOT padlock probe	/5Phos/TCGAGAATCTAAGCGACGATGATTACTGACTGCGTCTATTTACAAATATAATTGTGCAATGGTATCGACTCGAATGCAAAGAGCTTATAGCAA
Sequence-based reagent	gsb-n_PL3	This paper	SCRINSHOT padlock probe	/5Phos/CGTTGCCAGCCTTAATGATGATTACTGACTGCGTCTATTTACAAATATAATTGTGCAATGGTATCGACTCATGTTCATAATGCATGTATGGAAA
Sequence-based reagent	shark_PL1	This paper	SCRINSHOT padlock probe	/5Phos/TTCTCTGGTCAGAGTGCTCCTCTATGATTACTGACTGCGTCTATTTATTTCGTATTTCGCAAGCTGATCTTTTAGTATGGCTACTGCCTC
Sequence-based reagent	shark_PL2	This paper	SCRINSHOT padlock probe	/5Phos/AATTCTTCTACTGCCGTTTGTCCTCTATGATTACTGACTGCGTCTATTTATTTCGTATTTCGCAAGCTGATCTAGCTTGTAGTTGAGCAGG
Sequence-based reagent	shark_PL3	This paper	SCRINSHOT padlock probe	/5Phos/AACCGTTTGCACCAATTCTCCTCTATGATTACTGACTGCGTCTATTTATTTCGTATTTCGCAAGCTGATCTAATGGAAACTGGCTTAAATGTG
Sequence-based reagent	Marf1_PL1	This paper	SCRINSHOT padlock probe	/5Phos/GATTCGTCCAGCGAGTAGCTATGATTACTGACTGCGTCTATTTAATATATGTAATGGAACATTCCGATCCCAAGTAGAAAGCTGCGTATATGT
Sequence-based reagent	Marf1_PL2	This paper	SCRINSHOT padlock probe	/5Phos/TGAATAGGTCTTTATGTGCTGCTATGATTACTGACTGCGTCTATTTAATATATGTAATGGAACATTCCGATCCCGTTGGTCCGTTGTTAATAC
Sequence-based reagent	Marf1_PL3	This paper	SCRINSHOT padlock probe	/5Phos/TTGGAGTCCTTCCATTAAGGCTATGATTACTGACTGCGTCTATTTAATATATGTAATGGAACATTCCGATCCCGAATACTACAGTAAGGAACACTATG
Sequence-based reagent	cag_PL1	This paper	SCRINSHOT padlock probe	/5Phos/CCCTCTTTAATCGACTGTTTGCCTCTATGATTACTGACTGCGTCTATTTAAGTCTTCATCGCTCACTAGTAATGTTAAGTTCTCCACTCAACAGA
Sequence-based reagent	cag_PL2	This paper	SCRINSHOT padlock probe	/5Phos/ACGGGAGAACCAATCAAAGCCTCTATGATTACTGACTGCGTCTATTTAAGTCTTCATCGCTCACTAGTAATGGGTATATTCTCGGTACGGAC
Sequence-based reagent	cag_PL3	This paper	SCRINSHOT padlock probe	/5Phos/GCTTGGGTCAGGGAAGCCTCTATGATTACTGACTGCGTCTATTTAAGTCTTCATCGCTCACTAGTAATGGTATTTATATTGCACCTAAATTGCAT
Sequence-based reagent	CG7484_PL1	This paper	SCRINSHOT padlock probe	/5Phos/TCTGTGTTCCACTTGGTTATCTCTATGAT TACTGACTGCGTCTATTTAGATCAAATACGTAAGAGGACTGGATGAAGAACTCCTCGACAGTG
Sequence-based reagent	CG7484_PL2	This paper	SCRINSHOT padlock probe	/5Phos/ACTGAGGCTTGATGGTATCCTCTATGATTACTGACTGCGTCTATTTAGATCAAATACGTAAGAGGACTGGATAAAGTGCAGCATTGCTTAC
Sequence-based reagent	CG7484_PL3	This paper	SCRINSHOT padlock probe	/5Phos/GCGGATAGGCCCGCTCTATGATTACTGACTGCGTCTATTTAGATCAAATACGTAAGAGGACTGGATCTTTGAATAAAGGCCTGAATCT
Sequence-based reagent	Rab3GAP1_PL1	This paper	SCRINSHOT padlock probe	/5Phos/GAATGCAGTAGTGGAATTACTCCTCTATGATTACTGACTGCGTCTATTTAGACCGACTGATAAGCAAGGATTACAGCTAGTTTGAGTATTGCT
Sequence-based reagent	Rab3GAP1_PL2	This paper	SCRINSHOT padlock probe	/5Phos/CGTAGATCTGGACGAAGATTCCTCTATGATTACTGACTGCGTCTATTTAGACCGACTGATAAGCAAGGATTAGTATAGTAGTTCCATTTGGGAT
Sequence-based reagent	Rab3GAP1_PL3	This paper	SCRINSHOT padlock probe	/5Phos/GTGCAACAGTTGTCATTGTCCTCTATGATTACTGACTGCGTCTATTTAGACCGACTGATAAGCAAGGATTATCAGTCGTCGATTTACAATC
Sequence-based reagent	fz2_PL1	This paper	SCRINSHOT padlock probe	/5Phos/AGGAAAGTGTTTGTGGAAGTATGATCCTCTATGATTACTGACTGCGTCTATTTAGCGTTTGTTCTACCTGCCGTTTCTCAGTCTATTTCGTTG
Sequence-based reagent	fz2_PL2	This paper	SCRINSHOT padlock probe	/5Phos/TACAGGTAACATCCGATAACTATGTATGATCCTCTATGATTACTGACTGCGTCTATTTAGCGTTTGTTCTACCTGCCTCAAAGTAGGCTGCTTCG
Sequence-based reagent	fz2_PL3	This paper	SCRINSHOT padlock probe	/5Phos/TCAGTCGATTGTGTCTCATTTTATGATCCTCTATGATTACTGACTGCGTCTATTTAGCGTTTGTTCTACCTGCCCCCAGGATCAGGACCT
Sequence-based reagent	fz_PL1	This paper	SCRINSHOT padlock probe	/5Phos/ACTAAGAACTACGACTGCGCTATGATTACTGACTGCGTCTATTTACTTGTTCTACGAGTACTACAACTTTGATAGATGATGAACTTTATACAAAGCC
Sequence-based reagent	fz_PL2	This paper	SCRINSHOT padlock probe	/5Phos/AGATCGATATGGTGATGGGCTATGATTACTGACTGCGTCTATTTACTTGTTCTACGAGTACTACAACTTTGAGTCATGTTATATGGTATATTCTTGC
Sequence-based reagent	fz_PL3	This paper	SCRINSHOT padlock probe	/5Phos/GCTAAACTCTAAGTAACTTTCGTTACTATGATTACTGACTGCGTCTATTTACTTGTTCTACGAGTACTACAACTTTGAGCATAGGGAACGTCTATGT
Sequence-based reagent	fz4_PL1	This paper	SCRINSHOT padlock probe	/5Phos/TCATCGAGCCAAAGATCCCTATGATTACTGACTGCGTCTATTTACAATCCTCTACTATAACTTAATGCGGTCAAATAGCCGGAGATCAGAT
Sequence-based reagent	fz4_PL2	This paper	SCRINSHOT padlock probe	/5Phos/CGAAGAATACGAATTCGATACGCTATGATTACTGACTGCGTCTATTTACAATCCTCTACTATAACTTAATGCGGTCTGATTTGTTGTTCTTTCGTTC
Sequence-based reagent	fz4_PL3	This paper	SCRINSHOT padlock probe	/5Phos/GTCTAGAGTAGCGTCTCATTGCTATGATTACTGACTGCGTCTATTTACAATCCTCTACTATAACTTAATGCGGTGAAATGGTACCTAATCCAATTCC
Sequence-based reagent	fz3_PL1	This paper	SCRINSHOT padlock probe	/5Phos/GGTTATGTAGGAGGCCAGGATCCTCTATGATTACTGACTGCGTCTATTTACTCCAACTCTTTCGTATGCCACACAGGCTTAAATAGGAGGT
Sequence-based reagent	fz3_PL2	This paper	SCRINSHOT padlock probe	/5Phos/GTGGTTTACGTATTTCTCCGGATCCTCTATGATTACTGACTGCGTCTATTTACTCCAACTCTTTCGTATGCCATTTATATTACTGTGCGACTCTCA
Sequence-based reagent	fz3_PL3	This paper	SCRINSHOT padlock probe	/5Phos/GTCTTCTTCTGAGAGCTCGGATCCTCTATGATTACTGACTGCGTCTATTTACTCCAACTCTTTCGTATGCCACTAGAATGAGGGTCTCAGAC
Sequence-based reagent	spz_PL1	This paper	SCRINSHOT padlock probe	/5Phos/TGTTACTGTTGCCTCTTATTTTCTATGATTACTGACTGCGTCTATTTACGGTTATAAACGATATCAATATCGCGCCTTTATACTGGTAGCTGG
Sequence-based reagent	spz_PL2	This paper	SCRINSHOT padlock probe	/5Phos/CCACCGATCTTAAGTGTTTATAGTCTATGATTACTGACTGCGTCTATTTACGGTTATAAACGATATCAATATCGCGAAGCCCGATACCATCTG
Sequence-based reagent	spz_PL3	This paper	SCRINSHOT padlock probe	/5Phos/TCATTCCTCAAAGGACGAGTCTATGATTACTGACTGCGTCTATTTACGGTTATAAACGATATCAATATCGCGAGGGATTGTGCTCTTTAGTG
Sequence-based reagent	spz4_PL1	This paper	SCRINSHOT padlock probe	/5Phos/TCGTACTATTGGTATTTCCAGGTCTATGATTACTGACTGCGTCTATTTACAGACGATTTGCGATTGTATTGAAATCGCAGTTCCTTTAGTAGTACTTA
Sequence-based reagent	spz4_PL2	This paper	SCRINSHOT padlock probe	/5Phos/CAGGAGGGAATCTAATGGGTCTATGATTACTGACTGCGTCTATTTACAGACGATTTGCGATTGTATTGAAATCCTAATACTACTGTACAATTGTTCA
Sequence-based reagent	spz4_PL3	This paper	SCRINSHOT padlock probe	/5Phos/ATCGTTATTAGTCTAGTGATCGTCTATGATTACTGACTGCGTCTATTTACAGACGATTTGCGATTGTATTGAAATCAAATACCCGATAACCTCG
Sequence-based reagent	spz6_PL1	This paper	SCRINSHOT padlock probe	/5Phos/TCCTCTAAAGGCTAGAACTTAGATGATCCTCTATGATTACTGACTGCGTCTATTTAGACGATACCCTACCCAAGGCTTTCACCTCAAAGTTGTGT
Sequence-based reagent	spz6_PL2	This paper	SCRINSHOT padlock probe	/5Phos/GCTCGTTTGCTTCTTGTAGATGATCCTCTATGATTACTGACTGCGTCTATTTAGACGATACCCTACCCAAGGCACTAGACGATAATCCATCTCC
Sequence-based reagent	spz6_PL3	This paper	SCRINSHOT padlock probe	/5Phos/TTGTTTAGATCTCCGCAAATGATGATCCTCTATGATTACTGACTGCGTCTATTTAGACGATACCCTACCCAAGGGATTCTTGGGTATCTGACCC
Sequence-based reagent	spz5_PL1	This paper	SCRINSHOT padlock probe	/5Phos/AGGGTAAGACTAATGCTACTATGCCTCTATGATTACTGACTGCGTCTATTTACCCACACATGAAACACTCAATATCGATATCCATTAGATCGCTCAC
Sequence-based reagent	spz5_PL2	This paper	SCRINSHOT padlock probe	/5Phos/CGGCTTGTAGATTTGTTTGCCTCTATGATTACTGACTGCGTCTATTTACCCACACATGAAACACTCAATATCAATTCGGAAACCTAGTGTG
Sequence-based reagent	spz5_PL3	This paper	SCRINSHOT padlock probe	/5Phos/AGCGAGACATCGCGCCTCTATGATTACTGACTGCGTCTATTTACCCACACATGAAACACTCAATATCGATATCCAAGAGATCCATGTTG
Sequence-based reagent	ham_det1_FAM	This paper	SCRINSHOT detection probe	GTAGCTCCUGATCCTGTAUATCAAGAT/36-FAM/
Sequence-based reagent	ham_det2_FAM	This paper	SCRINSHOT detection probe	CCTTUAGAAAGCCAAAUGGTAGTTTC/36-FAM/
Sequence-based reagent	ham_det3_FAM	This paper	SCRINSHOT detection probe	CTTCCTTAUGTCATCGAAGUACTCC/36-FAM/
Sequence-based reagent	wnt2_det1_Cy3	This paper	SCRINSHOT detection probe	GATTAACAUAAATGCAGCUCATTAGTGAC/3Cy3Sp/
Sequence-based reagent	wnt2_det2_Cy3	This paper	SCRINSHOT detection probe	CTTCAGCAUGAGTCTAUCGCC/3Cy3Sp/
Sequence-based reagent	wnt2_det3_Cy3	This paper	SCRINSHOT detection probe	AACGCATTUCCTATGACAUAGCC/3Cy3Sp/
Sequence-based reagent	shg_det1_Cy5	This paper	SCRINSHOT detection probe	CCGCTCAAUAATTATCAAUCGAATCGAA/3Cy5Sp/
Sequence-based reagent	shg_det2_Cy5	This paper	SCRINSHOT detection probe	CGCACGAUTATCGCAUACGAT/3Cy5Sp/
Sequence-based reagent	shg_det3_Cy5	This paper	SCRINSHOT detection probe	ACTCCTCCUTGGTTACGUTGAT/3Cy5Sp/
Sequence-based reagent	shot_det1_Cy3	This paper	SCRINSHOT detection probe	TAGTTGGUGCATAGUCGCG/3Cy3Sp/
Sequence-based reagent	shot_det2_Cy3	This paper	SCRINSHOT detection probe	GTTTCCTCUAGATCCTTUAGGGTCTT/3Cy3Sp/
Sequence-based reagent	shot_det3_Cy3	This paper	SCRINSHOT detection probe	GAATTTCGUATTGAGTAUGCCATCGA/3Cy3Sp/
Sequence-based reagent	pbl_det	This paper	SCRINSHOT detection probe	GAATAUAAATGAGGUTGATGAUGAGGACT /FITC/
Sequence-based reagent	AP-2alpha_det	This paper	SCRINSHOT detection probe	CTCCUACACTCUTCTTAAGUACCTTTC /CY3/
Sequence-based reagent	CG11406_det	This paper	SCRINSHOT detection probe	GATAGUGGTCCACTGUCCTG /CY5/
Sequence-based reagent	AkhR_det	This paper	SCRINSHOT detection probe	CUGAGGAGAAUGATCATCGUGAT /FITC/
Sequence-based reagent	CG17186_det	This paper	SCRINSHOT detection probe	GAUAGAGGCTCUAGATTTCAUCTACG /CY3/
Sequence-based reagent	CG4872_det	This paper	SCRINSHOT detection probe	GAAACTAUCCCAAGGAGCUAGAAAT /CY5/
Sequence-based reagent	decay_det	This paper	SCRINSHOT detection probe	CUCAACCAGAUGAAGGAAAUGC /FITC/
Sequence-based reagent	PH4alphaMP_det	This paper	SCRINSHOT detection probe	CAATGTAUGGATTCGUGAACGC /CY3/
Sequence-based reagent	GstE11_det	This paper	SCRINSHOT detection probe	CTGGTGGAUGCCCGT /CY5/
Sequence-based reagent	Cog4_det	This paper	SCRINSHOT detection probe	CAATCAGUTCCTGAACUACAGTCA /FITC/
Sequence-based reagent	Gr64c_det	This paper	SCRINSHOT detection probe	GGCAAGGGAUACTTTCUGCT /CY3/
Sequence-based reagent	Idgf4_det	This paper	SCRINSHOT detection probe	GACUCAACGCAAACCUGTG /CY5/
Sequence-based reagent	CG17477_det	This paper	SCRINSHOT detection probe	GATAAUTACGGCUGGTCACUGT /CY3/
Sequence-based reagent	CG11069_det	This paper	SCRINSHOT detection probe	CAACTCTACUGATTGCCUACAACT /FITC/
Sequence-based reagent	AdSL_det	This paper	SCRINSHOT detection probe	CTCGAGUCGACGTTCTUGAT /FITC/
Sequence-based reagent	Tl_det	This paper	SCRINSHOT detection probe	CTACAACAAUAATCTGGUGCATGTG /CY3/
Sequence-based reagent	CG17283_det	This paper	SCRINSHOT detection probe	CAAGACCGAGUCAATCGTUAGA /FITC/
Sequence-based reagent	Hr46_det	This paper	SCRINSHOT detection probe	GAACATAAUCGAATTTGCUAAGCTCAUAC /CY3/
Sequence-based reagent	CG15117_det	This paper	SCRINSHOT detection probe	CAAACCCAUTATCATGTCUGAATAUGG /CY5/
Sequence-based reagent	gsb-n_det	This paper	SCRINSHOT detection probe	CAAATATAAUTGTGCAAUGGTATCGACUC /FITC/
Sequence-based reagent	shark_det	This paper	SCRINSHOT detection probe	TTTCGTATUTCGCAAGCUGATCT /CY3/
Sequence-based reagent	Marf1_det	This paper	SCRINSHOT detection probe	ATATATGUAATGGAACAUTCCGAUCCC /CY5/
Sequence-based reagent	cag_det	This paper	SCRINSHOT detection probe	AGTCTTCAUCGCTCACUAGTAATG /CY3/
Sequence-based reagent	CG7484_det	This paper	SCRINSHOT detection probe	GATCAAAUACGUAAGAGGACUGGAT /FITC/
Sequence-based reagent	Rab3GAP1_det	This paper	SCRINSHOT detection probe	GACCGACUGAUAAGCAAGGAUTA /CY3/
Sequence-based reagent	fz2_det	This paper	SCRINSHOT detection probe	GCGTTTGUTCTACCUGCC /FITC/
Sequence-based reagent	fz_det	This paper	SCRINSHOT detection probe	CTTGTTCUACGAGTACUACAACTUTGA /FITC/
Sequence-based reagent	fz4_det	This paper	SCRINSHOT detection probe	CAATCCTCUACTATAACUTAATGCGGT /CY3/
Sequence-based reagent	fz3_det	This paper	SCRINSHOT detection probe	CTCCAACUCTTTCGTAUGCCA /CY5/
Sequence-based reagent	spz _det	This paper	SCRINSHOT detection probe	CGGTTAUAAACGATAUCAATAUCGCG /CY5/
Sequence-based reagent	spz4_det	This paper	SCRINSHOT detection probe	CAGACGATUTGCGATUGTATUGAAAT /CY3/
Sequence-based reagent	spz6_det	This paper	SCRINSHOT detection probe	GACGAUACCCUACCCAAGG /CY5/
Sequence-based reagent	spz5_det	This paper	SCRINSHOT detection probe	CCCACACAUGAAACACUCAATATC /FITC/
Commercial assay or kit	SuperSignal West Femto Maximum Sensitivity Substrate	Thermo Fisher	Cat# 34096	
Commercial assay or kit	Effectene Transfection kit	QIAGEN	Cat# 301427	
Commercial assay or kit	Dual-Glo Luciferase Assay System	Promega	Cat# E2940	
