# Supplementary figures and images for: Drosophila hamlet mediates epithelial tissue assembly of the reproductive system
Source: eLife. 2025 Jul 4;13:RP104164. doi: 10.7554/eLife.104164 (PMC12227202; doi:10.7554/eLife.104164)

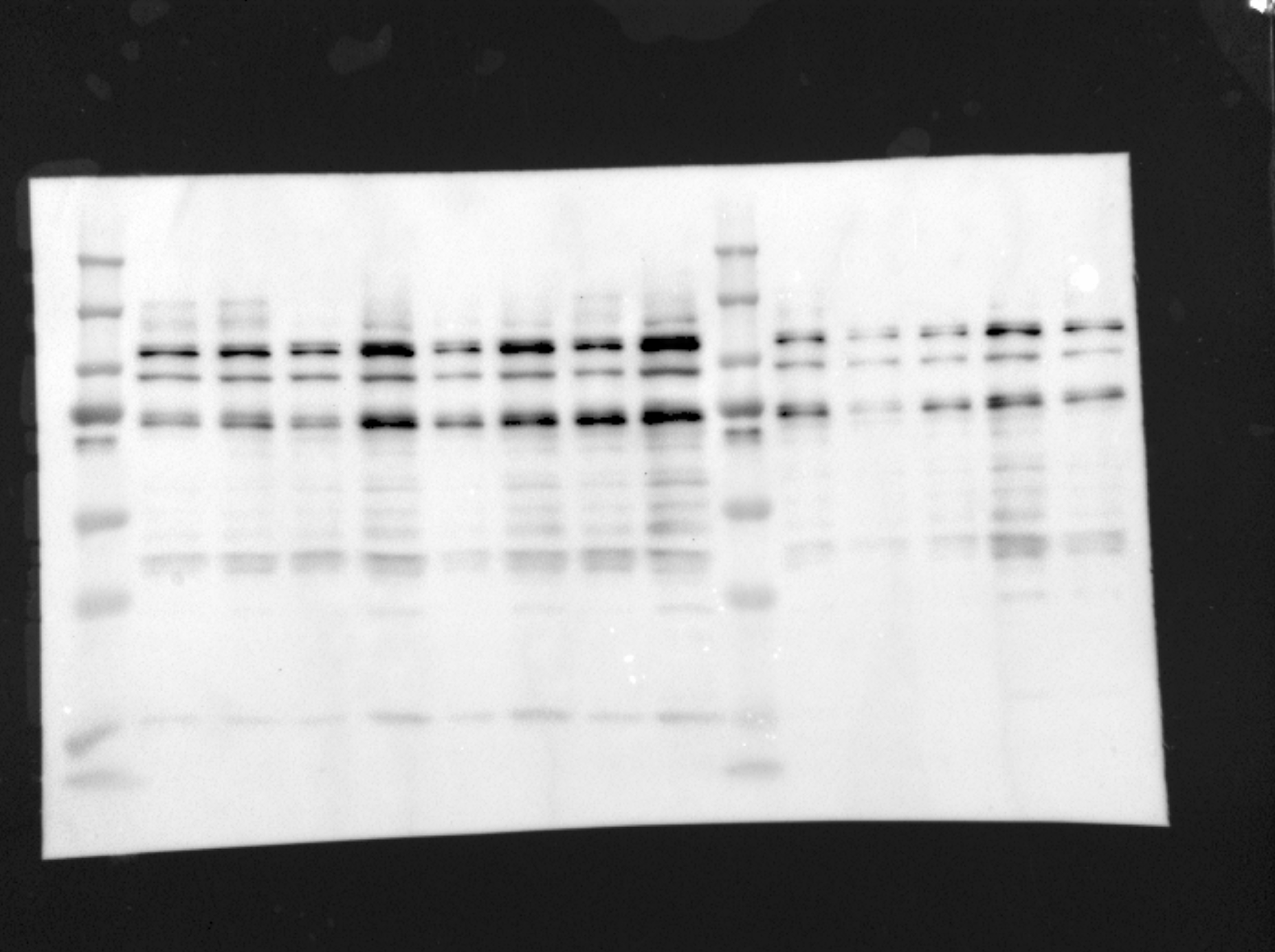

Supplement: Figure 1—figure supplement 1—source data 1. [file elife-104164-fig1-figsupp1-data1.zip › Figure1-figure supplement 1-source data1/Figure1-figure supplement 1C-Source Data1_raw.tif]

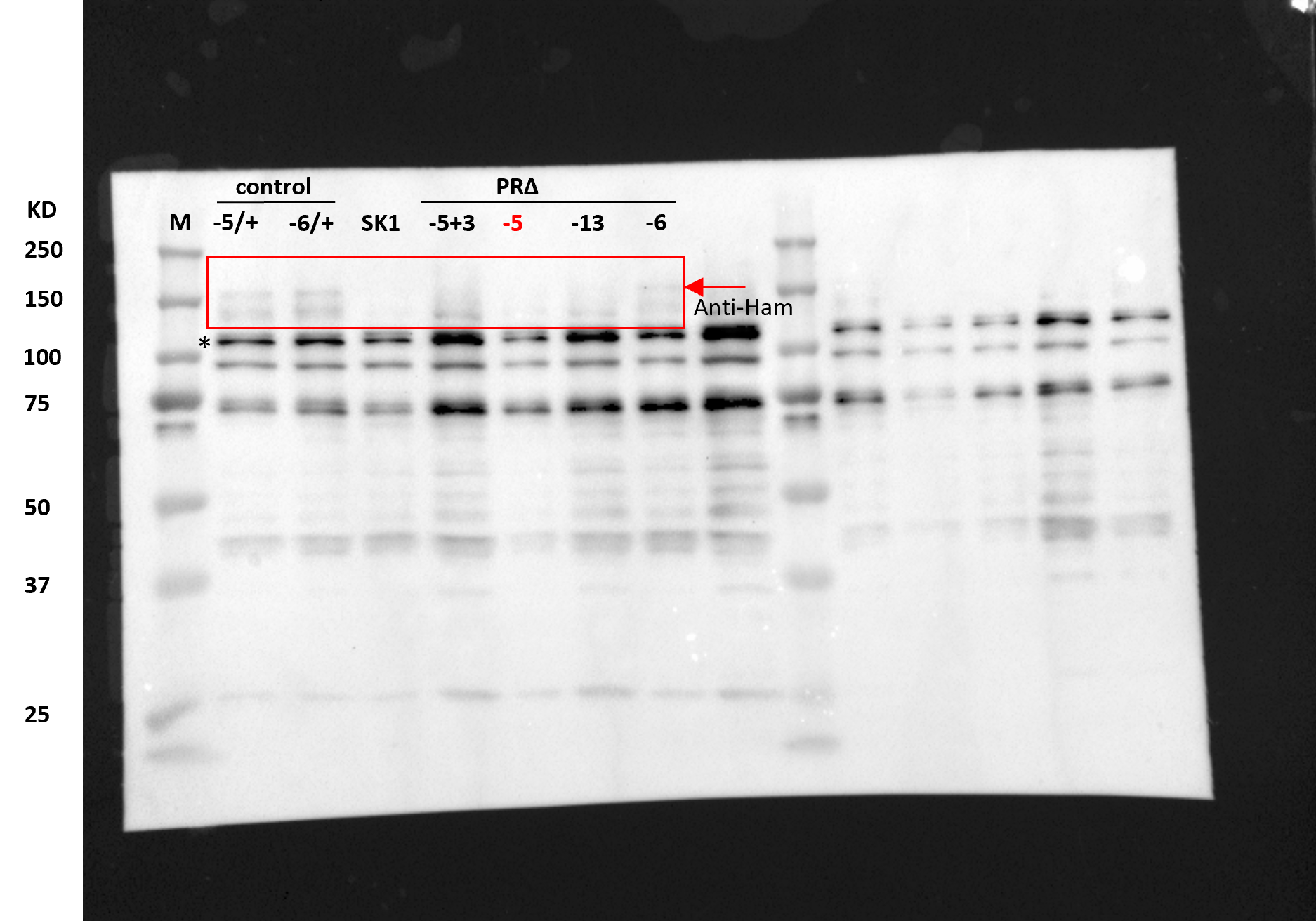

Supplement: Figure 1—figure supplement 1—source data 2. [file elife-104164-fig1-figsupp1-data2.zip › Figure1-figure supplement 1-source sata2/Figure1-figure supplement 1C-Source Data2_labelled.tif]
